# Chronic Cold Stress Alters the Skin Mucus Interactome in a Temperate Fish Model

**DOI:** 10.3389/fphys.2018.01916

**Published:** 2019-01-11

**Authors:** Ignasi Sanahuja, Laura Fernández-Alacid, Sergio Sánchez-Nuño, Borja Ordóñez-Grande, Antoni Ibarz

**Affiliations:** Departament de Biologia Cel.lular, Fisiologia i Immunologia, Universitat de Barcelona, Barcelona, Spain

**Keywords:** gilthead sea bream, low temperatures, mucus interactome, string analysis, zymography

## Abstract

Temperate fish are particularly sensitive to low temperatures, especially in the northern Mediterranean area, where the cold season decreases fish-farm production and affects fish health. Recent studies have suggested that the skin mucus participates in overall fish defense and welfare, and therefore propose it as a target for non-invasive studies of fish status. Here, we determine the mucus interactome of differentially expressed proteins in a temperate fish model, gilthead sea bream (*Sparus aurata*), after chronic exposure to low temperatures (7 weeks at 14°C). The differentially expressed proteins were obtained by 2D-PAGE of mucus soluble proteins and further assessed by STRING analyses of the functional interactome based on protein-protein interactions. Complementarily, we determined mucus metabolites, glucose, and protein, as well as enzymes involved in innate defense mechanisms, such as total protease and esterase. The cold mucus interactome revealed the presence of several subsets of proteins corresponding to Gene Ontology groups. “Response to stress” formed the central core of the cold interactome, with up-regulation of proteins, such as heat shock proteins (HSPs) and transferrin; and down-regulation of proteins with metabolic activity. In accordance with the low temperatures, all proteins clustered in the “Single-organism metabolic process” group were down-regulated in response to cold, evidencing depressed skin metabolism. An interactome subset of “Interspecies interaction between species” grouped together several up-regulated mucus proteins that participate in bacterial adhesion, colonization, and entry, such as HSP70, lectin-2, ribosomal proteins, and cytokeratin-8, septin, and plakins. Furthermore, cold mucus showed lower levels of soluble glucose and no adaptation response in total protease or esterase activity. Using zymography, we detected the up-regulation of metalloprotease-like activity, together with a number of fragments or cleaved keratin forms which may present antimicrobial activity. All these results evidence a partial loss of mucus functionality under chronic exposure to low temperatures which would affect fish welfare during the natural cold season under farm conditions.

## Introduction

Fish from temperate latitudes are typically exposed to broad fluctuations of water temperature. In nature, fish may use behavioral responses to overcome the threat that such fluctuations pose, through migration or by descending in the water column to take advantage of more stable temperatures. However, fish under aquaculture conditions cannot enact this natural behavior. When temperature variations approach certain upper or lower limits, according to the thermal tolerance range of the species, the consequences can be highly deleterious or even fatal. Both acute and chronic exposure to suboptimal temperatures generally have suppressive effects, particularly on adaptive immunity [reviewed in Abram et al. (2017)]. This has traditionally been assumed to be responsible for winter mortality in a large number of wild fish populations (Hurst, 2007). Furthermore, evidence has accumulated which suggests that diseases and handling disturbances in cultured species are also related to low water temperatures (Toranzo et al., 2005; Ibarz et al., 2010a). Gilthead sea bream have been cultured successfully for several decades and are an important species for the European aquaculture industry. However, they are particularly sensitive to low temperature, especially in the northern Mediterranean area, where cold affects fish health and decreases fish-farm production. A drop in temperature causes cold-induced fasting, thermal stress, and metabolic depression, resulting in a lower immune capacity and the fish being more susceptible to infection (Ibarz et al., 2010a). Moreover, in this species, there is no significant thermal compensation under sustained cold conditions and in such a situation any additional stress factors can cause fish to suffer metabolic collapse, even during cold recovery (Sánchez-Nuño et al., 2018a,b).

Management of fish farms is crucial to ensure fish health and welfare. Although potential stressors can be found at all stages of the production cycle, they are likely to be of greatest importance during the particularly sensitive period at low temperatures, during which fish are immunodepressed and suffer metabolic alterations (Tort et al., 1998a,b; Ibarz et al., 2010a; Silva et al., 2014). For this reason, analysis of the epidermal mucus has recently been proposed as a putative non-invasive and reliable method by which to study the response of fish physiology to environmental challenges (Benhamed et al., 2014; Sanahuja and Ibarz, 2015; Cordero et al., 2017; De Mercado et al., 2018; Fernández-Alacid et al., 2018, 2019). This method could replace other more invasive and deleterious diagnosis methods, such as hematological or histological analysis. In teleosts, the skin mucus is the first barrier against physical and chemical attacks. In addition to the structural mucin matrix, it contains components related to defense, metabolism, environmental influences and nutritional status (Esteban, 2012; Sanahuja and Ibarz, 2015). The skin mucus represents an important portal of pathogen entry, since it induces the development of biofilms and represents a favorable microenvironment for bacteria; the main disease agents in fish [reviewed in Benhamed et al. (2014)]. Skin mucus can trap and immobilize pathogens before they come into contact with epithelial surfaces, because it is impermeable to most bacteria and many pathogens (Mayer, 2003; Cone, 2009). Mucus is secreted by epidermal cells, mainly goblet cells, in a continuous effort to ensure its composition is adequate to prevent stable colonization by potentially infectious microorganisms as well as invasion by metazoan parasites (Ingram, 1980; Ellis, 2001; Nagashima et al., 2003). Thus, alterations in skin mucus due to low temperature conditions would modify this surface barrier and may facilitate bacterial adhesion, colonization, and entrance.

Therefore, the composition and characteristics of skin mucus are very important for the maintenance of its immune functions (Cone, 2009), as well as for other biological roles attributed to it: locomotion, respiration, ion regulation, excretion, and thermal regulation (Esteban, 2012). To extend the characterization of fish skin mucus, several studies have addressed the general mucosa proteome (Rajan et al., 2011; Guardiola et al., 2015; Sanahuja and Ibarz, 2015) and changes in skin mucus proteome in response to infections (Easy and Ross, 2009; Provan et al., 2013; Rajan et al., 2013). Fish mucus also serves as a repository of numerous innate immune factors; specific activities of enzymes, such as lysozyme, phosphatase, esterase, and protease also play an important role in mucosal immunity, which includes inhibitory or lytic activity against pathogens (Guardiola et al., 2014a). An interesting variety of protease families play important roles in mucus, such as serine and cysteine proteases, which are involved in organism defenses against bacteria and protozoa by lysing the parasite; or metalloproteases, which are involved in the activation of pro-cathepsin D, an enzyme that hydrolyses proteins for peptide production (Aranishi and Nakane, 1997; Cho et al., 2002b; Rakers et al., 2013). However, there is little information, at the level of skin mucus, on the role, and relevance of the activities of these proteases in cultured marine species, or their relationship with temperature fluctuations.

All this indicates the need to study the importance of mucus for overall fish defenses and welfare status during the problematic low-temperatures period of fish culture. Thus, the aim of the present work was to determine the main changes in the gilthead sea bream mucus interactome, based on protein–protein interactions, after chronic exposure to low temperatures (7 weeks at 14°C). The differentially expressed proteins were obtained by 2D-PAGE of soluble mucus proteins and further studied by STRING analysis of the functional interactome. The protease activities of skin mucus were also characterized by zymography, to identify different digestion bands. Our results therefore provide better understanding of mucus functionality at low temperatures in temperate marine species.

## Materials and Methods

### Animal Conditions

Gilthead sea bream, with an average body weight of 145 g, were obtained from a local fish farm and acclimated indoors at the facilities of the Faculty of Biology of the University of Barcelona (Barcelona, Spain) at 22°C for 2 weeks, using standard commercial fish feed (Skretting ARC). Following this period, the fish were randomly distributed into two groups in a water-recirculating system. The system was composed of 400 L tanks with solid and biological filters. Water temperature and oxygen concentration were monitored, while nitrite, nitrate, and ammonia concentrations were maintained at initial levels throughout the experimental period. For the experiment, the fish were initially maintained at 22°C for 4 weeks, after which time mucus samples were obtained non-invasively from 12 animals (Warm), and thereafter the water temperature was cooled to 14°C over 5 days (at 1.5°C per day) and maintained at this temperature the remained of a total 7 weeks period (including the 5 days cooling down period). At the end of this period, mucus samples were obtained from 12 animals (Cold). For both samplings, warm and cold, fish were 24 h-fasted. All animal-handling procedures were conducted following the European Union Council (86/609/EU) and Spanish and Catalan government-established norms and procedures and with Ethics and Animal Care Committee of the University of Barcelona approval (permit no. DAAM 9383).

To collect mucus samples, fish were lightly anesthetized with 2-phenoxyethanol (100 ppm, Sigma-Aldrich) to avoid stress of the manipulation. Sterile glass slides were used to carefully remove mucus from the over-lateral line from the front in the caudal direction, as explained in Fernández-Alacid et al. (2018). The sterile glass was gently slid along both sides of the animal and the epidermal mucus was carefully pushed into a sterile tube (2 mL). Non-desirable areas of the operculum, and ventral-anal and caudal fins were avoided. The mucus collected was immediately frozen with liquid nitrogen and stored at −80°C until analysis.

### Two-Dimensional Electrophoresis of Mucus Samples

#### Protein Extraction

Mucus samples for two-dimensional electrophoresis (2D-PAGE) protocols were solubilized in an equal volume of ice-cold lysis buffer (4 mL · g^−1^ tissue; 7 M urea; 2 M thiourea, 2% w/v CHAPS; and 1% protease inhibitor mixture) and centrifuged at 20,000 g for 15 s at 4°C, with the resultant supernatant aliquoted, avoiding pellet resuspension, and surface lipid layer. The supernatants obtained were subjected to a clean-up procedure (ReadyPrep 2-D clean-up kit, BioRad, Alcobendas, Spain) to enhance protein extraction, as previously described in Sanahuja and Ibarz (2015), and the proteome map of soluble skin mucus proteins was obtained by 2D-PAGE. The significantly expressed proteins were further analyzed by LC-MS/MS and identified using database retrieval. Protein concentration was determined by the Bradford assay with bovine serum albumin (BSA) as standard (BioRad).

#### Dimensional Electrophoresis Separation

Two mucus samples were polled in order to obtain 450 μg of protein dissolved in 450 μL of rehydration buffer containing 7 M urea, 2 M thiourea, 2% w/v CHAPS, 0.5% v/v IPG buffer, 80 mM DTT, and 0.002% bromophenol blue. Five such samples of skin mucus protein extract from each condition (Warm and Cold) were loaded onto 24 cm, pH 3-10 NL IPG strips (GE Healthcare, Madrid, Spain). Isoelectric focusing was performed using an IPGhor instrument (Amersham Biosciences, Stockholm, Sweden), following the manufacturer's instructions (active rehydration at 50 V for 12 h followed by a linear gradient from 500 to 8,000 V, at 48,000 V · h^−1^). The focused strips were equilibrated in two steps as follows: 15 min with equilibration buffer I (65 mM DTT, 50 mM Tris-HCl, 6 M urea, 30% glycerol, 2% SDS, and bromophenol blue) and then 15 min with equilibration buffer II (135 mM iodoacetamide, 50 mM Tris-HCl, 6 M urea, 30% glycerol, 2% SDS, and bromophenol blue). Equilibrated strips were set directly onto 12.5% polyacrylamide gels, sealed with 0.5% w/v agarose, and separated at a constant voltage of 50 V for 30 min followed by 200 V for about 6 h, until the blue dye reached the bottom of an Ettan DALT II system (Amersham Biosciences). Proteins were fixed for 1 h in methanol: acetic acid, 40:10, and stained overnight using colloidal Coomassie Brilliant Blue G-250. Gel staining was removed by consecutive washing steps with distilled water until the best visualization was achieved.

#### Gel Image Analysis

Gels stained with Coomassie Brilliant Blue were scanned in a calibrated Imagescanner (BioRad) and digital images captured using Quantity-One software (BioRad). The images were saved as uncompressed TIFF files. Gel images were analyzed using the software package ImageMaster 2D, version 6.01 (GE Healthcare). Proteins were detected using the automated routine of the ImageMaster 2.0 software, combined with manual editing when necessary to remove artifacts. The background was removed, and normalized volumes were calculated as follows: the volume of each protein spot was divided by the total volume of all the protein spots included in the analysis. Normalized protein spot values were used to select the 300 most abundant proteins in each condition to be further analyzed for their differential expression.

#### Protein Digestion

Protein in-gel trypsin was digested manually (sequencing grade modified, Promega). Selected spots with differential expression were manually cut out from reference gels and were washed sequentially with 25 mM ammonium bicarbonate (NH_4_HCO_3_) and acetonitrile (ACN). The proteins were reduced with 20 mM DTT solution for 60 min at 60°C and alkylated with a 50 mM solution of iodine acetamide for 30 min at room temperature. After sequential washings with buffer and acetronitrile, the proteins were digested overnight at 37°C with 80 ng of trypsin. Peptides were extracted from the gel matrix with 10% formic acid (FA) and can, pooled and dried in a vacuum centrifuge. The trypsin-digested peptide samples were analyzed by LC-MS/MS.

#### LC-MS/MS Analysis

Dry-down peptide mixtures were analyzed in a nanoAcquity liquid chromatographer (Waters, Cerdanyola del Vallés, Spain) coupled to an LTQ-Orbitrap Velos (Thermo Scientific, Barcelona, Spain) mass spectrometer. Trypsin digests were resuspended in 1% FA solution and an aliquot was injected into chromatographic separation equipment. The peptides were trapped in a Symmetry C18TM trap column (5, 180 μm × 20 mm, Waters), and were separated using a C18 reverse-phase capillary column (ACQUITY UPLC M-Class Peptide BEH column; 130 Å, 1.7, 75 μm × 250 mm, Waters). The gradient used for the elution of the peptides was 1 to 40% B in 20 min, followed by 40 to 60% in 5 min (A: 0.1% FA; B: 100% CAN, 0.1% FA), with a 250 nL · min^−1^ flow rate. Eluted peptides were subjected to electrospray ionization in an emitter needle (PicoTipTM, New Objective, Woburn. MA, USA) with an applied voltage of 2,000 V. Peptide masses (m/z 300–1,700) were analyzed in data dependent mode where a full Scan MS was acquired in the Orbitrap with a resolution of 60,000 FWHM at 400 m/z. Up to the 10th most abundant (minimum intensity of 500 counts) peptides were selected from each MS scan and then fragmented in the linear ion trap using CID (38% normalized collision energy) with helium as the collision gas. The scan time settings were: Full MS: 250 ms (1 microscan) and MSn: 120 ms. The.raw data files generated were collected with Thermo Xcalibur (v.2.2).

#### Database Search

The.raw files obtained in the mass spectrometry analysis were used to search the public database Uniprot Actinopterygii (v.23/3/17). A database containing common laboratory contaminant proteins was added to this database. The software used was Thermo Proteome Discoverer (v1.4.1.14) with Sequest HT as the search engine. The following search parameters were applied: 2 missed cleavage sites as well as fixed and variable modifications; carbamidomethyl of cysteine and oxidation of methionine, respectively. Peptide tolerance was 10 ppm and 0.6 Da for MS and MS/MS spectra, respectively. Both a target and a decoy database were searched in order to obtain a false discovery rate (FDR), and thus estimate the number of incorrect peptide–spectrum matches that would exceed a given threshold. The results were filtered so only proteins identified with at least 2 high confidence (FDR > 1%) peptides were included in the lists.

#### Interactome Analysis

Gene Ontology (GO) enrichment analysis was performed with the UniProt-IDs of identified proteins retrieved from UniProt knowledgebase (UniProtKB). The UniProt-IDs were submitted to PANTHER (www.pantherdb.org) to cluster the proteins into different groups related to their biological process, according to GO annotation (GO terms). Only results with *p* < 0.05 were accepted. The interactome was derived from confidence analysis of the protein–protein interaction network by the STRING Program v10.5.

### Biochemical Parameters

Before mechanical homogenization, the scales collected in mucus samples were individually removed. Mucus samples were diluted (v/v) with Milli-Q water to extract the mucus adhered to the scales. The mechanical homogenization was performed by a sterile Teflon stick to desegregate the mucus mesh before centrifugation at 14,000 g. The resultant mucus supernatants were collected avoiding the surface lipid layer, aliquoted, and stored at −80°C.

Glucose concentration was determined by an enzymatic colorimetric test (LO-POD glucose, SPINREACT^®^, Girona, Spain). Briefly, glucose oxidase (GOD) catalyzes the oxidation of glucose to gluconic acid. The hydrogen peroxide (H_2_O_2_) formed is detected by a chromogenic oxygen acceptor phenol, 4–aminophenazone (4-AP), in the presence of peroxidase (POD). Following the manufacturer's instructions for plasma determination, but with slight modifications, 10 μL of mucus extracts or standard solutions (from 0 to 100 mg · dL^−1^) were mixed in triplicate with 200 μL of working reagent and incubated for 10 min at 37°C. The OD was determined at λ = 505 nm with a microplate reader (Infinity Pro200 spectrophotometer, Tecan, Barcelona, Spain). The glucose values were expressed as μg glucose · mL^−1^ of skin mucus.

The protein concentration of the homogenized mucus was determined using the Bradford assay (Bradford, 1976) with BSA as standard (Sigma). Mucus samples or standard solutions (from 0 to 1.41 mg · mL^−1^) were mixed in triplicated with 250 μL of the Bradford reagent and incubated for 5 min at room temperature. The OD was determined at λ = 596 nm with a microplate reader (Infinity Pro200 spectrophotometer, Tecan). The protein values were expressed as mg protein · mL^−1^ of skin mucus.

Esterase activity was determined according to the method of Ross et al. (2000). Equal volumes of skin mucus and 0.4 mM p-nitrophenyl myristate substrate in 100 mM ammonium bicarbonate buffer containing 0.5% Triton X-100 (pH 7.8, 30°C) were incubated. The OD was continuously measured at 1 min intervals over 3 h at 405 nm in a plate reader. The initial rate of the reaction was used to calculate the activity. One unit of activity was defined as the amount of enzyme required to release 1 mmol of p-nitrophenol product in 1 min. Enzyme activity was measured as mIU · mg^−1^ of protein.

Total alkaline protease activity (TPA) was spectrophotometrically measured in the homogenates following Moyano et al. (1996). Thus, the samples first reacted in 50 mM Tris-HCl pH 9.0 buffer containing 1% casein. After 30 min, the reaction was stopped by adding trichloroacetic acid (TCA, 12%). The samples were then maintained for 1 h at 4°C and centrifuged (7500 g, 5 min, 4°C). Supernatant absorbance was measured at 280 nm. Each sample was analyzed in triplicate and individual blanks were established by adding TCA solution before the homogenate. Bovine trypsin was used as the standard. Enzyme activity was measured as IU · mg^−1^ of protein.

Lysozyme activity was measured according to the turbidimetric method described by Parry et al. (1965) with some modifications. One hundred ml of skin mucus diluted 1/2 with 10 mM PBS, pH 6.2, was placed in flat-bottomed 96-well plates in triplicate. To each well, 100 ml of freeze-dried *Micrococcus lysodeikticus* (0.3 mg · ml^−1^, Sigma) was added as a lysozyme substrate. The reduction in absorbance at 450 nm was measured after 0 and 15 min at 22°C in a plate reader. One unit of lysozyme activity was defined as a reduction in absorbance of 0.001 min^−1^. The units of lysozyme present in skin mucus were obtained from a standard curve made with hen egg white lysozyme (HEWL, Sigma). Enzyme activity was measured as mIU · mg^−1^ of protein.

### Zymography

Individual alkaline protease activities were also studied using zymograms according to the method established in fish by Santigosa et al. (2008) and modified by García-Meilán et al. (2013). Briefly, 30 μg of mucus protein was diluted and loaded on 12% polyacrylamide gel. Electrophoresis was performed at a constant current of 15 mA per gel for 90 min (Bio Rad Mini PROTEAN Tetra Cell, 4°C). Protease-active fractions were visualized using the method described by García-Carreño et al. (1993) where the gels were incubated at 4°C under agitation in Tris-HCl 50 mM pH 8.2 solution containing 2% casein. After 30 min, the temperature was raised to room temperature for 90 min with shaking. The gels were washed and stained in a methanol:acetic:water solution (40:10:40) with 0.1% of Coomassie Brilliant Blue R-250 (Bio-Rad). Destaining was carried out using the same solution without colorant until the right visualization of the digested bands was achieved. Pure trypsin was used as a positive control. To determine the molecular weight of protease fractions, a commercial weight marker was used (RPN 800E, GE Healthcare). The gels were further scanned in an ImageScanner III (Epson J181A) and caseinolytic bands were identified. Total protein was normalized using the Quantity One software (Bio-Rad) including total lane intensity. Negative images from each sample were captured to show the intensity for the corresponding caseinolytic band. The relative digestion units for each band were obtained by the relation between the band quantification (from the negative image) and the total lane intensity (previously removing the background). Digestion band intensity was calculated as arbitrary units of casein digestion capacity: the area intensity of each specific digested band, via the negative image, was related to the total intensity of the respective undigested lane, see Supplementary Material for detailed information.

### Western Blot

Mucus samples were centrifuged at 12,000 g for 10 min and the protein concentration in the supernatant measured. Supernatants were treated with Laemmli loading buffer and 30 μg of proteins resolved on SDS-polyacrylamide (10%) gels and transferred to nitrocellulose. Membranes were then blocked overnight (depending on the antibody affinity) with 4% Non-Fat Dry Milk (BioRad) in Tris-buffered saline (TBS) (pH 7.4) containing 0.05% (w/v) Tween 20 (TTBS). Membranes were washed three times in TTBS and probed for 1 h with the following primary antibodies: anti-cytokeratin-8 (Thermo-Scientific) and anti-actin (Sigma-Aldrich). Detection was performed with an adequate HRP-conjugated IgG (Santa Cruz Biotechnology, Heidelberg, Germany). The blots were visualized with enhanced chemiluminescence (Clarity from Bio-Rad) and detected and scanned on a Fujifilm LAS-3000 Imager (Fujifilm Corporation, Tokyo, Japan). Digital images were quantified using Quantity One software (BioRad) and normalized by the total amount of protein detected by Ponceau S staining (Sigma-Aldrich).

### Statistical Analysis

Metabolite amounts, enzyme activities, zymography, and Western blot comparison between Warm and Cold were analyzed by Student's *t*-test. Proteins (spots) that were found to vary in abundance between the Warm and Cold samples were analyzed for significance using Student's *t*-test. The Shapiro-Wilk test was first used to ensure the normal distribution of the data, while the uniformity of the variances was determined by Levene's test. All statistical analysis was undertaken with commercial software (PASW version 21.0, SPSS Inc., Chicago, IL, USA). The STRING databases were used to obtain direct protein–protein interactions (PPI), the interactome, by the search tool for the retrieval of interacting genes/proteins STRING Program v10.5 (Szklarczyk et al., 2017). The selected stat indicators were the “clustering coefficients” and “PPI enrichment *p*-value,” which correspond to a measure of how connected the nodes in the network are, and the “count in gene set” which indicates the number of proteins included and their “False discovery rate.” The enrichment tests, from STRING software, are done for a variety of classification systems (Gene Ontology, KEGG, Pfam and InterPro), and employ a Fisher's exact test followed by a correction for multiple testing (Benjamini and Hochberg, 1995; Rivals et al., 2007).

## Results

### Mucus Proteome

The aim of our mucus proteome analysis was to determine the differentially expressed proteins in skin mucus by comparing the “Warm” mucus proteome and “Cold” mucus proteome at the end of the extended period at 14°C. More than 1,200 protein spots were detected in the mucus proteome of all the samples. Of these spots, 20 were down-regulated and 32 were up-regulated due to the chronic cold (master gel with labeled spots is shown in Supplementary Figure 1). Table 1 shows the mass spectrometry characterization of the differentially expressed spots, followed by MASCOT database searches which yielded their theoretical pI and molecular weight, and established probable protein identity. The table also shows the observed molecular weight and pI, in accordance with its location in the 2D gel. Most of the proteins identified correspond to protein sequences that have already been reported in teleost species, except for three spots which correspond to structural proteins that show the greatest homologies to distinct species of mammals.

**Table 1:** Identification of the 52 differentially expressed proteins by cold in gilthead sea bream epidermal mucus grouped by Structural, Metabolic, or Protective functions.

**Spot^a^**	**INT^b^**	**SEM^b^**	**INT^c^**	**SEM^c^**	**T-Stu^d^**	**INT^d^**	**Protein identity^e^**	**Accession n${}^{0_{e}}$**	**Gene^f^**	**Theoretical^e^**		**Observed^e^**		**Peptides^e^**	**SQ^e^**	**Species^e^**	**Gene^f^**	**UniprotKB^f^**
**ID**	**(%)**		**(%)**			**FOLD**		**(gI)**	**symbol**	**MW**	**pI**	**MW**	**pI**	**matched**	**(%)**		**number**	
1	0.38	0.003	0.53	0.048	0.026	1.39	Transferrin	327243044	TF	74.23	6.30	75.00	7.11	38/(52)	79	*Sparus aurata*	7018	P02787
2	0.31	0.010	0.43	0.043	0.044	1.37	Transferrin	327243042	TF	76.10	5.90	72.00	6.10	17/(35)	30	*Sparus aurata*	7018	Q90YH6
5	0.35	0.009	0.17	0.021	0.001	0.49	Deoxycytidylate deaminase	FG590567	DCTD	13.80	6.80	11.00	4.40	1/(1)	12	*Sparus aurata*	1635	P32321
6	0.28	0.014	0.37	0.026	0.014	1.33	Stress protein HSC70-1	212274295	HSPA8	71.50	5.20	66.00	4.40	14/(23)	26	*Seriola quinqueradiata*	3312	P11142
15	0.03	0.004	0.25	0.071	0.026	7.56	40S ribosomal protein like 12	47224253	RPS12	14.40	7.24	12.00	7.25	10/(10)	64	*Tetraodon nigroviridis*	6206	P25398
26	0.15	0.007	0.12	0.011	0.048	0.76	Coactosin-like	85719983	COTL1	10.00	5.50	11.00	4.60	1/(1)	10	*Ictalurus punctatus*	23406	Q14019
38	0.26	0.011	0.21	0.013	0.021	0.81	Coactosin-like	47221902	COTL1	16.20	4.90	11.00	4.00	4/(8)	22	*Tetraodon nigroviridis*	23406	Q14019
44	0.15	0.019	0.23	0.010	0.005	1.50	Heat shock protein A1	47223819	HSPA1A	71.40	5.20	66.00	4.30	11/(15)	19	*Tetraodon nigroviridis*	3303	P08107
47	0.05	0.003	0.19	0.036	0.028	4.02	Hnrpa01 protein	323649982	HNRNPA1	13.71	6.39	18.00	6.34	2/(8)	39	*Perca flavescens*	3178	P09651
56	0.13	0.004	0.08	0.004	0.000	0.66	Proliferating cell nuclear antigen	430721599	PCNA	28.66	4.72	26.00	4.07	9/(9)	58	*Dicentrarchus labrax*	5111	P12004
71	0.04	0.005	0.13	0.026	0.033	2.99	Periplakin-like	551527179	PPL	205.96	6.18	76.00	6.02	1/(9)	5	*Xiphophorus maculatus*	5493	O60437
82	0.03	0.008	0.09	0.008	0.001	2.91	Keratin. type II cytoskeletal 8-like	551498795	KRT8	53.45	5.01	54.00	4.49	5/(15)	30	*Xiphophorus maculatus*	3856	P05787
94	0.05	0.005	0.13	0.012	0.000	2.53	Epiplakin	221047999	EPPK1	30.75	4.84	39.00	6.40	3/(5)	25	*Epinephelus coioides*	83481	P58107
97	0.02	0.003	0.16	0.031	0.006	7.13	Peptidyl-tRNA hydrolase	317418901	PTRHD1	20.20	4.63	16.00	3.97	3/(3)	26	*Dicentrarchus labrax*	391356	Q6GMV3
98	0.03	0.003	0.13	0.023	0.008	4.14	Keratin 12	528509044	KRT12	49.81	5.35	15.00	3.97	3/(11)	17	*Danio rerio*	3859	Q99456
111	0.02	0.002	0.05	0.010	0.040	2.45	60S ribosomal protein	11095761	RPL23A	10.91	9.70	15.00	8.09	3/(3)	29	*Oncorhynchus mykiss*	6147	P62750
119	0.11	0.008	0.13	0.007	0.037	1.24	Protein disulfide-isomerase-like	475653184	PDIA3	55.87	5.60	54.00	4.89	13/(18)	31	*Dicentrarchus labrax*	2923	P30101
121	0.10	0.018	0.05	0.006	0.048	0.46	Alpha 2 globin	99122203	HBA2	15.83	8.72	12.00	4.11	8/(8)	52	*Sparus aurata*	3040	P69905
124	0.16	0.006	0.12	0.014	0.037	0.72	Myosin light polypeptide 6	229366002	MYL6	17.00	4.50	11.00	3.80	5/(7)	36	*Anoplopoma fimbria*	4637	P60660
126	0.04	0.014	0.11	0.016	0.023	2.42	Keratin. type II cytoskeletal 5-like	P13647	KRT5	62.34	7.74	76.00	6.67	16/(27)	43	Mammal sps.	3852	P13647
133	0.06	0.008	0.11	0.015	0.026	1.75	Transferrin	327243044	TF	74.23	6.30	75.00	4.74	27/(27)	45	*Sparus aurata*	7018	P02787
134	0.18	0.011	0.11	0.020	0.026	0.63	Malate dehydrogenase mitochondrial	410905057	MDH2	35.80	8.60	32.00	8.10	10/(16)	37	*Takifugu rubripes*	4191	P40926
140	0.18	0.023	0.12	0.011	0.032	0.64	Esterase D	348524078	ESD	31.60	5.90	31.00	5.20	3/(6)	14	*Oreochromis niloticus*	2098	P10768
144	0.11	0.005	0.15	0.011	0.042	1.36	Betaine homocysteine M-transferase	388260758	BHMT	44.07	6.71	43.00	8.39	13/(18)	64	*Sparus aurata*	635	Q93088
152	0.17	0.024	0.09	0.017	0.018	0.51	Translation initiation factor 5A	47209413	EIF5A	17.50	5.20	14.00	4.40	3/(15)	13	*Tetraodon nigroviridis*	1984	P63241
154	0.11	0.008	0.21	0.036	0.029	1.99	Heat shock 70 kDa protein 1-like	410933029	HSPA1L	52.50	5.24	16.00	6.93	2/(4)	11	*Takifugu rubripes*	3305	P34931
155	0.24	0.029	0.14	0.015	0.016	0.59	Keratin type I cytoskeletal 13	229366514	KRT13	49.72	5.36	17.00	4.14	3/(17)	27	*Anoplopoma fimbria*	3860	P13646
159	0.18	0.006	0.10	0.012	0.002	0.59	Inorganic pyrophosphatase-like	432903493	PPA1	33.40	5.10	33.00	4.50	6/(9)	23	*Oryzias latipes*	5464	Q15181
160	0.07	0.006	0.15	0.021	0.016	2.24	Inositol monophosphatase 1-like	583999941	IMPA1	27.26	5.30	27.00	5.12	11/(12)	57	*Neolamprologus brichardi*	3612	P29218
163	0.20	0.009	0.09	0.013	0.000	0.43	14-3-3 protein zeta/delta	10719663	YWHAZ	28.10	4.70	24.00	4.10	2/(9)	7	*Fundulus heteroclitus*	7534	P63104
167	0.04	0.009	0.13	0.021	0.008	3.45	Keratin. type I cytoskeletal 13	229366514	KRT13	48.48	5.33	20.00	3.94	3/(15)	22	*Anoplopoma fimbria*	3860	P13646
169	0.03	0.004	0.16	0.034	0.014	4.74	Keratin. type II cytoskeletal 5-like	573882490	KRT5	61.05	5.41	13.00	4.50	4/(20)	25	*Lepisosteus oculatus*	3852	P13647
170	0.03	0.002	0.10	0.015	0.006	3.98	Intermediate filament protein ON3	551498797	ION3	58.55	5.48	12.00	4.75	2/(15)	22	*Xiphophorus maculatus*	N/A	P18520
176	0.13	0.014	0.08	0.011	0.023	0.64	Proteasome subunit alpha type-6-like	410916067	PSMA6	27.40	6.35	23.00	6.96	23/(23)	63	*Takifugu rubripes*	5687	P60900
177	0.14	0.018	0.08	0.012	0.027	0.60	UBQ-like modifier-activating enzyme	432865628	UBA1	117.97	5.76	96.00	4.51	3/(8)	11	*Oryzias latipes*	7317	P22314
181	0.02	0.004	0.12	0.029	0.023	5.77	F-type lectin 2	334883514	Rb-FTL2	34.53	6.34	27.00	6.34	2/(2)	9	*Oplegnathus fasciatus*	N/A	F7J049
184	0.04	0.001	0.10	0.023	0.034	2.72	β-actin	6693629	ACTB	41.81	5.48	42.00	7.49	3/(24)	65	*Pagrus major*	60	P60709
189	0.03	0.003	0.14	0.019	0.002	5.11	Keratin. type II cytoskeletal 5-like	18858425	KRT5	58.55	5.41	13.00	4.74	4/(17)	19	*Danio rerio*	3852	P13647
190	0.25	0.022	0.12	0.021	0.002	0.48	Gelsolin	FM026536	GSN	85.90	5.90	77.00	6.90	2/(3)	6	*Dicentrarchus labrax*	2934	P06396
192	0.06	0.002	0.10	0.008	0.006	1.82	β-actin	261286856	ACTB	40.83	5.83	12.00	7.00	3/(3)	10	*Anguilla japonica*	60	P60709
193	0.05	0.007	0.09	0.012	0.025	1.84	Keratin. type II E3-like protein	48476437	N/A	38.60	4.96	13.00	4.28	11/(20)	46	*Sparus aurata*	N/A	Q4QY72
197	0.02	0.004	0.08	0.019	0.035	3.36	Peptidyl-prolyl cis-trans isomerase F	348508637	PPIF	21.02	8.94	13.00	9.04	2/(3)	17	*Oreochromis niloticus*	10105	P30405
199	0.16	0.009	0.09	0.008	0.001	0.58	Protein disulfide-isomerase-like	498926878	PDIA3	57.40	4.60	52.00	3.60	6/(11)	11	*Maylandia zebra*	2923	P30101
201	0.03	0.006	0.08	0.015	0.021	2.53	Septin-2-like	551486665	SEPT2	40.03	6.28	36.00	6.26	4/(16)	66	*Xiphophorus maculatus*	4735	Q15019
205	0.12	0.004	0.08	0.016	0.039	0.65	UMP-CMP kinase-like	348500565	CMPK1	24.90	8.60	20.00	6.80	2/(7)	11	*Oreochromis niloticus*	51727	P30085
206	0.05	0.009	0.09	0.011	0.018	1.91	Keratin. Type II cytoskeletal 1	P04264	KRT1	65.98	8.12	14.00	9.32	18/(21)	38	Mammal sps.	3848	P04264
207	0.18	0.036	0.08	0.013	0.040	0.42	Periplakin-like	499048295	PPL	184.00	5.90	98.00	7.00	7/(5)	4	*Maylandia zebra*	5493	O60437
213	0.03	0.006	0.10	0.019	0.011	3.68	Keratin. Type II cytoskeletal 1	P04264	KRT1	65.98	8.12	16.00	8.72	30/(34)	49	Mammal sps.	3848	P04264
234	0.14	0.014	0.08	0.014	0.030	0.61	Aldo-keto reductase family	432941989	AKR1B10	35.62	6.46	31.00	7.54	1/(7)	18	*Oryzias latipes*	57016	O60218
236	0.04	0.003	0.07	0.010	0.012	2.00	Transferrin	327243044	TF	74.23	6.30	74.00	4.73	19/(19)	35	*Sparus aurata*	7018	P02787
247	0.15	0.020	0.08	0.006	0.008	0.52	Malate dehydrogenase	551491925	MDH1	38.40	7.60	34.00	7.10	6/(15)	18	*X. maculatus*	4190	P40925
251	0.03	0.003	0.07	0.013	0.020	2.55	Keratin. type I cytoskeletal 13	212995	KRT13	49.72	5.36	14.00	4.76	2/(11)	16	*Carassius auratus*	3860	P13646

a *Spot number from Supplementary Figure 1 and the corresponding spot ID in Tables 1, 2*.

b *Mean and standard error of the mean (SEM) for each individual spot from 5 replicate Warm condition gels (pools of soluble protein extract from 2 or 3 fish)*.

c *Mean and standard error of the mean (SEM) for each individual spot from 5 replicate Cold condition gels (pools of soluble protein extract from 2 or 3 fish)*.

d *Statistic Student T-test (p < 0.05) and intensity fold for each individual spot from 5 replicate gels*.

e *Protein identities, accession number, theoretical, and observed MW and pI, peptides matched (unique peptides), percentage sequence coverage (SQ) and species identification were supplied by the Mascot Search Results (Matrix science). Further details of search conditions in Material and Methods section*.

f *Gene symbol, gene number (Entrez gene database from NCBI, http://www.ncbi.nlm.nih.gov/), and UniprotKB (http://www.uniprot.org) of each protein were obtained from the Genecards database search process (http://www.genecards.org). The UniprotKB number was used for further Gene Ontology enrichment analysis in Table 2*.

**Figure 1:**
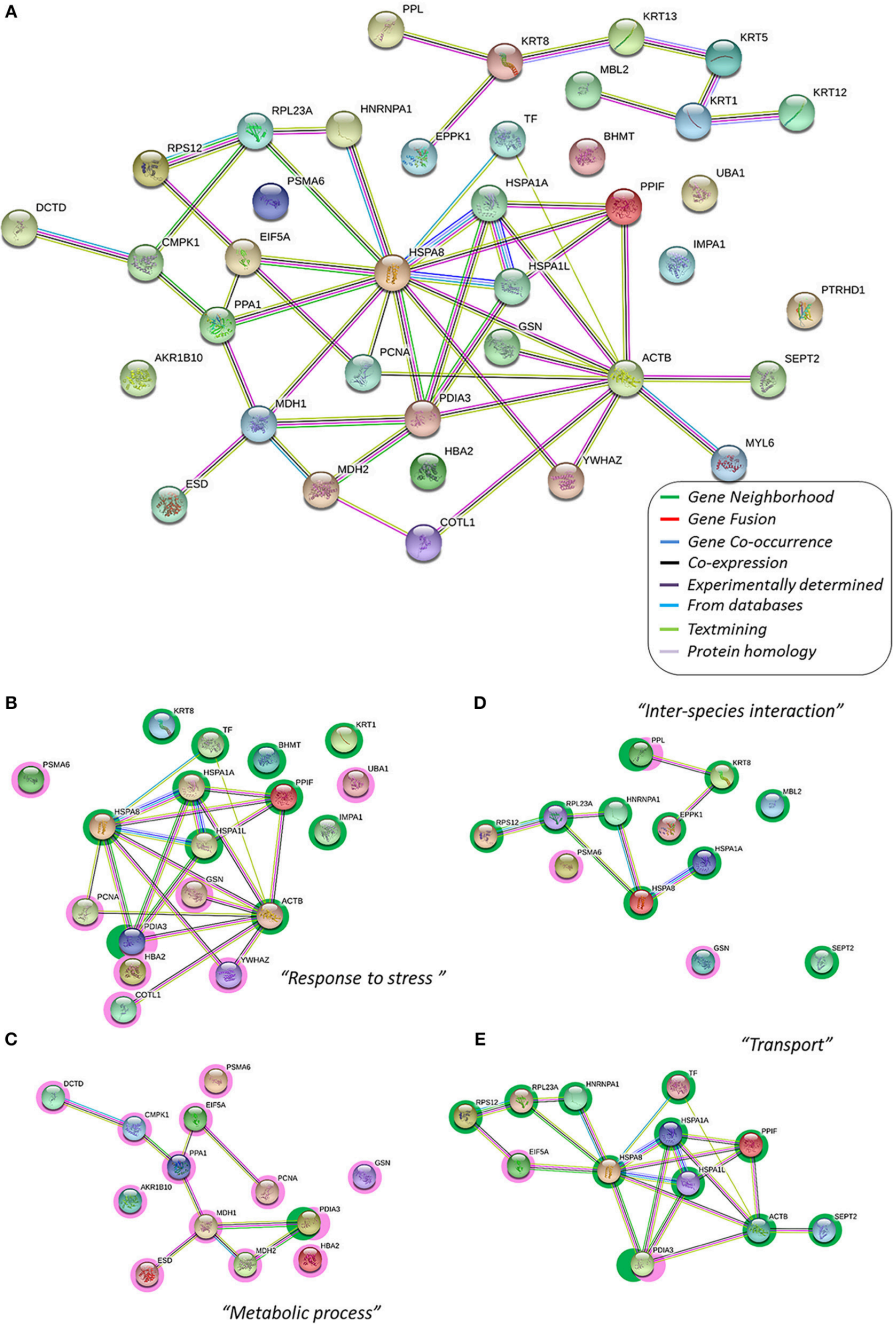
The protein–protein interaction network, the interactome, of gilthead skin mucus proteins differentially expressed by chronic low temperatures. In this network, nodes are proteins, lines represent the predicted functional associations, and the color of the lines represents the strength of the predicted functional interactions between the proteins, according to the STRING databases (Szklarczyk et al., 2017). **(A)** Total protein interactome; all protein listed in Table 2 have been included to obtain the network. Relevant data from the network stats (such as the clustering coefficient and the PPI enrichment *p*-value) are provided in Supplementary Table 1. **(B–E)** Main Gene Ontology clusters obtained by GO-enrichment groups with significance (see Table 2), where green shaded nodes correspond to proteins that are up-regulated by chronic cold stress and pink shaded nodes corresponded to down-regulated proteins due to chronic cold stress. Each sub-cluster have been performed using the protein groups from Table 2. Relevant data from the network stats and the functional enrichment process are also provided in Supplementary Table 1.

The proteins identified were clustered, firstly according their main function as: structural-, metabolic- or protective-related proteins. Accordingly, Table 2 summarizes the name of the proteins belonging to each GO group; only six proteins could not be directly grouped. The proteins were also analyzed using the “cellular component GO,” for their specific location. Forty-six of the fifty-two proteins (Table 2) belong to the “extracellular vesicular exosome” (GO: 0070062, *p* = 1.43e-37) indicating that all these proteins could be released into the extracellular region directly via exosomal vesicles. Moreover, the STRING databases were used to obtain direct protein–protein interactions (Figure 1A).

**Table 2:** Regulation and biological process aggrupation of differentially expressed proteins sorted by Structural, Metabolic, or Protective functions.

**Spot ID^a^**	**Protein identity^b^**	**Gene^c^ symbol**	**Regulation^d^**		**Biological process groups**				**Exosome^e^**
					**Response^e^ to stress**	**Metabolic^e^ process**	**Transport^e^**	**Interspecies^e^ interaction**	
			**UP-**	**DOWN-**					
**PROTECTIVE PROTEINS**									
1, 2, 133, 236	Transferrin	TF	*		X		X		X
6	Stress protein HSC70-1	HSPA8	*		X		X	X	X
44	Heat shock protein A1	HSPA1A	*		X		X	X	X
154	Heat shock 70 kDa protein 1-like	HSPA1L	*		X		X		X
181	F-type lectin 2	MBL-2	*					X	-
119, 199	Protein disulfide-isomerase-like	PDIA3	*	*	X	X	X		X
121	Alpha 2 globin	HBA2		*	X	X			X
177	Ubiquitin-like modifier-activating enzyme 1-like	UBA1		*	X				X
163	14-3-3 protein zeta/delta	YWHAZ		*	X				X
140	Esterase D	ESD		*		X			X
**METABOLIC PROTEINS**									
15	40S ribosomal protein like 12	RPS12	*				X	X	-
111	60S ribosomal protein	RPL23A	*				X	X	X
47	Heterogeneous nuclear ribonucleoprotein A1	HNRNPA1	*				X	X	X
197	Peptidyl-prolyl cis-trans isomerase F	PPIF	*		X		X		-
160	Inositol monophosphatase 1-like	IMPA1	*		O				X
144	Betaine homocysteine M-transferase	BHMT	*		O				X
97	Peptidyl-tRNA hydrolase	PTRHD1	*						X
56	Proliferating cell nuclear antigen	PCNA		*	X	X			X
176	Proteasome subunit alpha type-6-like	PSMA6		*	X	X		X	X
152	Translation initiation factor 5A	EIF5A		*		X			X
5	Deoxycytidylate deaminase	DCTD		*		X			X
247	Malate dehydrogenase	MDH1		*		X			X
134	Malate dehydrogenase mitochondrial	MDH2		*		X			X
159	Inorganic pyrophosphatase-like	PPA1		*		X			X
205	UMP-CMP kinase-like	CMPK1		*		X			X
234	Aldo-keto reductase family 1 member B10-like	AKR1B10		*		X			X
**STRUCTURAL PROTEINS**									
170	Intermediate filament protein ON3-like	ION3	*				X		-
201	Septin-2-like isoform X2	SEPT2	*				X	X	X
94	Epiplakin-like protein	EPPK1	*					O	-
193	Keratin, type II E3-like protein	N/A	*						-
206, 213	Keratin, Type II cytoskeletal 1	KRT1	*		X				X
82, 251	Keratin, type II cytoskeletal 8-like	KRT8	*		X			X	X
184, 192	β-actin	ACTB	*		X		X		X
126, 169, 189	Keratin, type II cytoskeletal 5-like	KRT5	*						X
98	Keratin 12 isoform X1	KRT12	*						X
155, 167	Keratin, type I cytoskeletal 13	KRT13	*	*					X
71, 207	Periplakin-like	PPL	*	*				O	X
124	Myosin light polypeptide 6	MYL6		*					X
26,38	Coactosin-like	COTL1		*	X				X
190	Gelsolin-S1/S2-like	GSN		*	X	X		X	X

a *Spot number from Supplementary Figure 1 (where green spots are over-expressed and pink spots were under-expressed) and the corresponding spot ID in Tables 1, 2*.

b *Protein identities were supplied by the Mascot Search Results (Matrix science). Further details of search conditions in Material and Methods section*.

c *Gene symbol of each protein were obtained from the Genecards database search process (http://www.genecards.org)*.

d *Up- or Down- protein regulation in cold condition. The intensities of each protein and statistical analysis Student T-test (p < 0.05) are shown in Table 1*.

e *Classification of proteins into different categories based on Gene Ontology enrichment analysis (GO) using UniprotKB number (shown in Table 1). Related to Biological process GO: Response to stress (GO:0006950, p = 7.05e-06); Single-organism metabolic process (GO:0044710, p = 3.85e-02); Transport (GO:0006810, p = 2.39e-02); Interspecies interaction between organisms (GO:0044419, p = 2.22e-05). An additional cluster of Cellular component categories has been added: Extracellular vesicular exosome (GO:0070062, p = 1.43e-37)*.

The resulting Cold-mucus interactome has a central core of differentially expressed proteins (18 different proteins) related to the biological process “Response to stress” (GO:0006950, *p* = 7.05e-06, Figure 1B; Table 2). This group clustered together 11 over-expressed proteins. Some were associated with a protective role, such as four spots identified as transferrin (TF, spots 1, 2, 133, 236), three different heat shock proteins (HSP8, spot 6; and HSPA1, spots 44, and 154) and a lectin-type form (MBL-2, spot 181). Others were associated with matrix structure functions, such as β-actin (ACTB, spots 184, 192) and keratins (KRT8, spots 82 and 251; and KRT1, spots 206 and 213). While yet others were associated with other stress-related proteins and enzymatic activity (PDIA3, spot 118; PPFI, spot 197; IMPA 1, spot 160; and BHMT, spot 144). This group also included seven under-expressed proteins: three with a protective role (HBA2, spot 121; UBA1, spot 177; and YWHAZ, spot 163), two with metabolic activity (PCNA, spot 56; and PSMA6, spot 176), and two actin-related proteins, gelsolin (GSN, spot 190) with actin-assembly regulatory function and coactosin (COTL1, spots 26, 38) with actin filament-stabilizer function.

The second most significant group of protein interactions, namely “Single-organism metabolic process” (GO:0044710, *p* = 3.85e-02), included thirteen proteins that are under-expressed at low temperatures (Figure 1C). Most of these proteins showed enzymatic activities: related to lipid metabolism, such as an esterase (ESD, spot 140) and an inorganic pyrophosphatase (PPA1, spot 159); enzymes required for cellular nucleic acid biosynthesis, a deaminase (DCTD, spot 5) and a kinase (CMPK1, spot 206); and other activities, such as proteasomal (PSMA6, spot 176), malate dehydrogenases (MDH1, spot 247; and MDH2, spot 134), and an oxidoreductase (AKR1, spot 234). The “Transport” group (GO:0006810, *p* = 2.39e-02, Figure 1D) represents biological processes related to the directed movement of substances into, out of or within a cell, or between cells. This group included 11 proteins modified in the mucus interactome; all over-expressed, indicating a putative increased response at low water temperatures of skin mucus exudation. They belong to protective functions (HSPs and PDIA3), to structural functions of intermediate filaments (ION3, spot 170; and SEPT2, spot 201), to ribosomal activity (RPS12, spot 15; and RPL23A, spot 111), and to protein folding (PTRHD1, spot 97; and HNRNPA1, spot 47). Finally, a number of proteins was grouped within the biological process “Interspecies interaction between organisms” (GO:0044419, *p* = 2.22e-05, Figure 1E). This GO group clustered together seven over-expressed proteins (HSPA8, HSPA1A, KRT8, RPS12, RPL23A, HNRNPA1, and SEPT2) and two under-expressed proteins (GSN and PSMA6). Moreover, other proteins that were over-expressed were also related to this process of species interaction at the extracellular matrix level, such as lectin (MBL2, spot 181), a carbohydrate-binding protein, and two proteins in the plakin structures of the skin barrier function: epiplakin (EPPK1, spot 94) and periplakin (PPL, spots 71 and 207).

### Biochemical Parameters and Mucus Zymography

Levels of soluble glucose and soluble protein in skin mucus were also obtained before and after the 7 weeks cold challenge at 14°C. We then calculated the glucose/protein ratio individually to normalize putative mucus dilution during the sampling process (data in Table 3). The present study revealed that skin mucus glucose exudation was greatly affected by the cold challenge: a 5-fold reduction from 15.9 ± 2.0 to 3.4 ± 0.4 μg · mL^−1^ of mucus extract (*p* < 0.05). However, the amount of soluble mucus protein was not modified by the cold challenge. As a result, the glucose/protein ratio was reduced by 6-fold, evidencing different affectation of glucose and protein exudation capacity. As regards the enzymatic activities of total protease (TPA), esterase and lysozyme, all related to the immune response, they showed no cold compensation via increased presence in mucus at the end of cold period: values of TPA were around 1.6-1.7 (IU · mg protein^−1^) and esterase activity was around 0.6 (IU · mg protein^−1^); whereas lysozyme activity was not detectable under the current analytical conditions.

**Table 3:** Metabolites and enzymatic parameters of epidermal mucus after a cold challenge.

	**Warm**	**Cold**
Glucose (μg/mL)	14.1 ± 0.8	3.4 ± 0.4^*^
Protein (mg/mL)	14.4 ± 0.5	15.4 ± 1.5
Glucose/Protein ratio (μg/mg)	0.97 ± 0.1	0.22 ± 0.0^*^
Total protease activity (IU/mg pr)	1.6 ± 0.3	61.6 ± 0.9
Esterase (mIU/mg pr)	0.56 ± 0.04	0.60 ± 0.01
Lysozyme (IU/mg pr)	n.d	n.d

*Values are mean ± SEM from pools of 2 fish (n = 6). Asterisks indicate significant differences between Warm and Cold conditions (p < 0.05; Student's T-test). N.d, no detected*.

**Figure 2:**
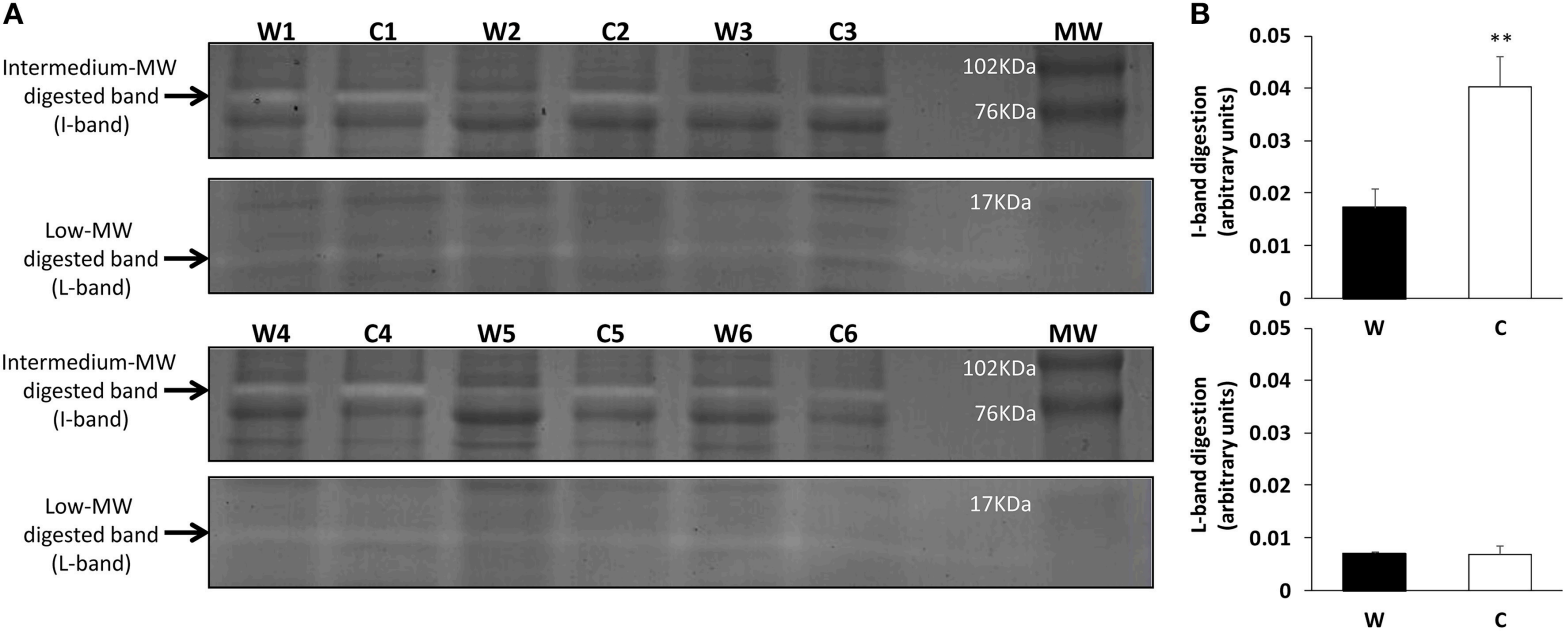
Zymograms of skin mucus protease activities of warm (W) and cold challenged (C) gilthead sea bream. **(A)** Gel zymography: electrophoresis was performed on polyacrylamide (12% acrylamide) gels. Two clear digested bands were appreciated and quantified. To determine the molecular weight of the protease fractions, a commercial weight marker was used (MW-lane). The gels were cut to simplify interpretation (intact gels are provided as Supplementary Figure 2). **(B)** Intermediate band relative intensity **(C)** Low band relative intensity. Both the I-band and L-band intensity were calculated as arbitrary units of trypsin digestion capacity (see Supplementary Figure 2 for detailed information). ^**^ indicates significant differences (*p* < 0.01; Student's *t*-test).

To characterize the alkaline protease activity pattern of sea bream skin mucus, zymographic analysis was performed using casein digestion activity for the first time in skin mucus of this species. The resulting zymograms (Figure 2A) show the presence of two digested bands with caseinolytic activity at the molecular weights of 12–15 kDa (low MW-band or L-band) and 76–80 kDa (intermediate MW-band or I-band). Enlarged gel images are provided as Supplementary Figure 2. Individual activities were calculated for both the I-band and the L-band (Figures 2B,C, respectively) measuring the intensity of each specific digested band, via a negative image, and then normalizing by the total intensity of the respective undigested lane (details of negative image evaluation are provided in Supplementary Figure 2). Although total protease measured spectrophotometrically was not affected by cold challenge, the zymography study revealed that the caseinolytic activity of the specific I-band increased 5-fold in response to the chronic exposure to low temperature.

### Identification of Protein Fragments With Putative Antimicrobial Activity

**Figure 3:**
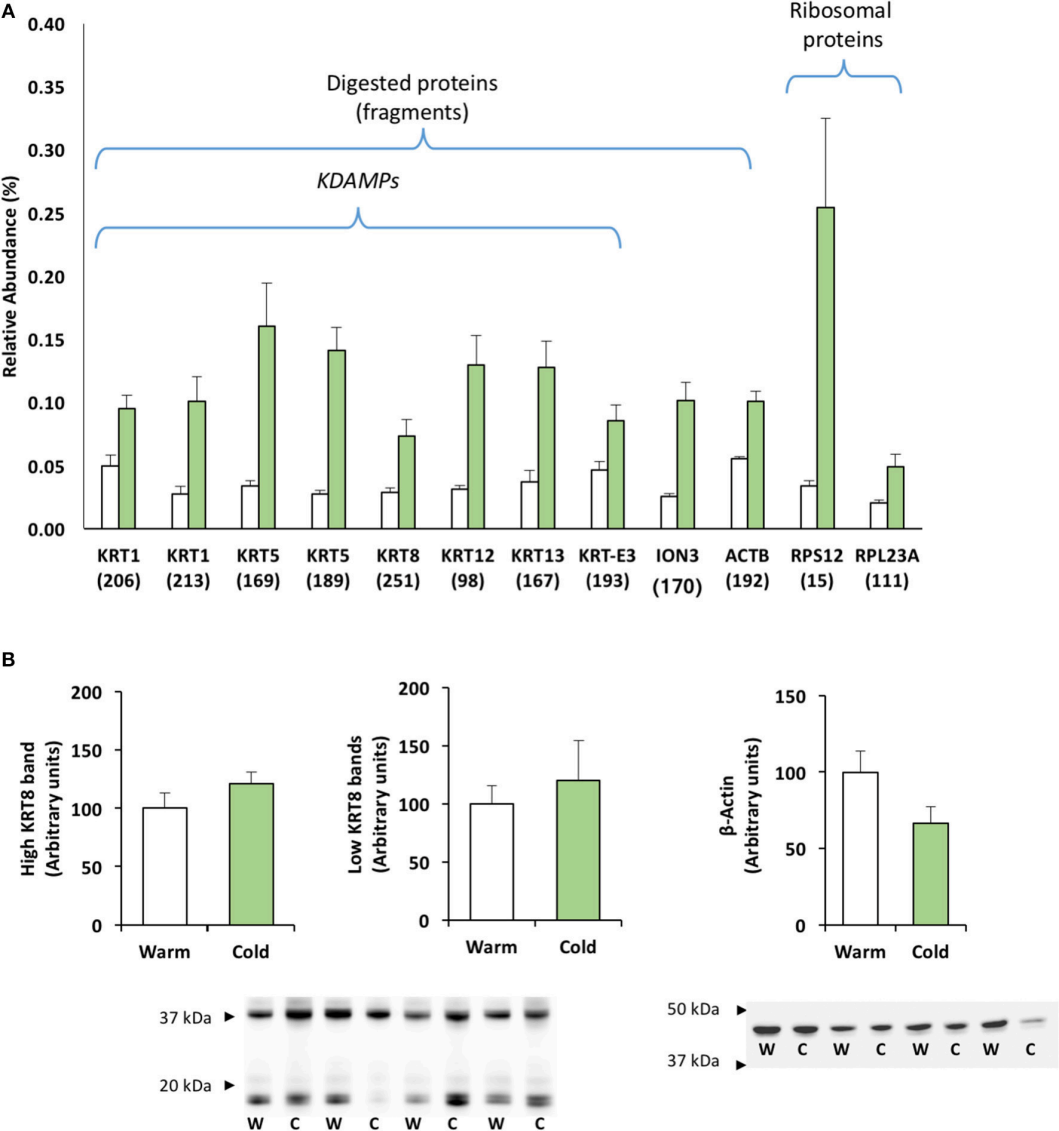
Relative expression of identified protein fragments with putative antimicrobial activity. **(A)** Histogram of protein abundance. Values corresponded to mean ± S.E.M. of the relative abundance of differentially expressed proteins. The digested proteins corresponded to proteins identified with observed MW lower than theoretical MW (see details in Table 1). Over-expressed ribosomal proteins are shown due to their antimicrobial activity. **(B)** Cytokeratin-8 (KRT8) and β-actin relative abundances by Western blot analysis.

The study of proteins that were significantly expressed by 2D-PAGE revealed a number of proteins located at a lower molecular weight (Observed MW) than expected (Theoretical MW); they are plotted in Figure 3A. Ten of these proteins correspond to different keratin fragments, so-called “KDAMPs” (keratin-derived antimicrobial peptides), all of which were significantly over-expressed (Figure 3A). Two spots identified as KRT1 had observed MWs of 14 and 16 kDa, instead of the theoretical 66 kDa (data in Table 1); two spots identified as KRT5 had observed MWs of 13 kDa, instead of the theoretical 61 kDa; one spot identified as KRT8, spot 251, had an observed MW of 14 kDa, instead of the theoretical 50 kDa; one spot identified as KRT12, spot 98, had an observed MW of 15 kDa, instead of the theoretical 50 kDa; one spot identified as KRT13, spot 167, had an observed MW of 20 kDa, instead of the theoretical 49 kDa; and one spot identified as KRT-E3, spot 193, had an observed MW of 13 kDa instead of the theoretical 39 kDa. Besides keratin fragments, two additional structural proteins were identified as protein fragments with lower MWs: ION3, spot 170, and ACTB, spot 192, with observed MWs of around 12 kDa. Figure 3A also includes the relative abundance of two ribosomal proteins, related to putative antimicrobial activity (see the Discussion section): 40S ribosomal protein (RPS12, spot 15) and 60S ribosomal protein (RPL23A, spot 111) increased 7.5- and 2.5-fold in sea bream mucus at low temperatures.

Finally, Figure 3B shows the Western blot analysis of cytokeratin-8 and β-actin, to compare with the proteome data. At least two clear bands appeared for cytokeratin-8: at 40 kDa, with no coincidence with significantly increased spots of KRT8; and at 14 kDa, coinciding with the KRT8 fragment (spot 251) reported above, with a possible extra band at 20 kDa. However, neither Western blot band was significantly over-expressed. For β-actin, a single band appeared at around 45–48 kDa, corresponding to the expected MW; however, no lower MW bands were observed which could have matched with the actin fragment (ACTB, spot 192) observed in the proteome.

## Discussion

Monitoring and reporting the general health status and welfare of fish is an important issue for fish farms. With the aim of combining the search for biomarkers with a non-invasive method, here for the first time we studied the skin mucus proteome of gilthead sea bream subjected to low temperatures for an extended period. We combined the valuable screening of differentially expressed proteins in the mucus proteome with the evaluation of some innate defenses, such as TPA, and esterase and lysozyme activity. In addition, skin mucus metabolites, glucose, and protein were analyzed as new indicators of fish welfare (in accordance with Fernández-Alacid et al., 2018, 2019) and mucus zymography was characterized, as it is classically performed on gut mucosa (Alarcón et al., 1998; Santigosa et al., 2008).

The amounts of soluble glucose and protein in skin mucus have recently been proposed as non-invasive markers of fish responses to stress challenges, together with mucus lactate and cortisol levels (Cordero et al., 2017; De Mercado et al., 2018; Fernández-Alacid et al., 2018, 2019). The drastic reduction in soluble glucose exuded after 50 days of low temperature exposure would seem to indicate a chronic condition of low-energy availability, as is also true for glucose plasma values during cold-associated reduced ingesta (Ibarz et al., 2010b; Sánchez-Nuño et al., 2018a). Whereas, soluble mucus glucose was reduced by a half in response to 2 weeks of deprivation at warm temperatures (Fernández-Alacid et al., 2018), here, the sustained low-temperature condition reduced mucus glucose 5-fold. The lower levels of glucose exudation not only indicated energy sparing but would seem to be associated with a compromised state at low temperatures. The importance of maintaining soluble carbohydrates in fish mucus has been reported, because bacteria adhesion correlates negatively with carbohydrate-rich mucus constituents and positively with lipid- and protein-rich mucus constituents (Tkachenko et al., 2013).

Fish epidermal mucus serves as a repository of numerous innate immune response protein components, playing roles in inhibitory or lytic activity against different types of pathogens, such as glycoproteins, lysozyme, complement proteins, C-reactive protein, flavoenzymes, proteolytic enzymes, and antimicrobial peptides (Guardiola et al., 2014a,b; Sanahuja and Ibarz, 2015). Among these, the most commonly characterized have been proteases, lysozyme and esterases. In response to low temperatures, neither TPA nor esterase activity changed. This is in contrast to reported increased activities when fish are exposed to pathogens, stress or environmental factors, such as salinity (Easy and Ross, 2009; Caruso et al., 2011; Jung et al., 2012; Loganathan et al., 2013). In addition, we can expect the functionality of these enzymes to be temperature dependent, with activity reduced at 14°C compared to 22°C. Thus, the same amount of enzyme at lower temperatures would mean weakened defenses during the cold season, due to a lack of cold adaptation, as has repeatedly been reported for sea bream metabolism during the cold season (Vargas-Chacoff et al., 2009; Ibarz et al., 2010b; Silva et al., 2014; Sánchez-Nuño et al., 2018a,b). With regard to lysozyme activity, we detected no mucus activity, in spite of it having been reported in several species including sea bream (Guardiola et al., 2014b).

The release of proteases into skin mucus may act directly on a pathogen or may prevent pathogen invasion indirectly by modifying mucus consistency to increase the sloughing of mucus and thereby the removal of pathogens from the body surface (Aranishi et al., 1998). The zymographic evaluation in the current study, comparing warm and cold caseinolytic activity, showed two well-defined bands at MWs of ~12–15 kDa (L-band) and 76–80 kDa (I-band). This demonstrates for the first time the presence of different protease activities in sea bream skin mucus. The L-band in the zymography matched trypsin-like activity: a low-molecular-weight serine protease with strong bactericidal activity against Gram positive bacteria, which has been observed in the skin mucus of rainbow trout (Hjelmeland et al., 1983), Atlantic salmon (Braun et al., 1990; Ross et al., 2000), and olive flounder (Jung et al., 2012). Meanwhile, the I-band matched reported activity of metalloproteases in the skin mucus of Atlantic salmon (Firth et al., 2000) and several freshwater species (Nigam et al., 2012). In higher vertebrates, metalloprotease production has been associated with response to injury, disease or inflammation (Woessner, 1991), activating various immune factors, such as cytokines, chemokines, receptors (McCawley and Matrisian, 2001), other proteases like cathepsines, and antimicrobial peptides (Cho et al., 2002a,b). Interestingly, the cold challenge increased those particular activities 5-fold in gilthead sea bream, reflecting differences between mucus protease properties according to stressor. The existence of trypsin-like serine proteases has been considered to play an important role in innate immunity, on top of its digestive function [reviewed in Esteban (2012)]. However, low temperatures did not alter the L-band activity of sea bream mucus, indicating, as with TPA, the lack of cold adaptation of trypsin-like activities. Further studies are needed of the specific role of skin mucus proteases and environmental challenges in fish.

The mucus proteome has been shown to be a powerful tool to devise putative bioindicators of fish welfare and physiological status via non-invasive methods in several fish species, such as Atlantic cod (Rajan et al., 2011), lumpsucker (Patel and Brinchmann, 2017), discus (Chong et al., 2005), European sea bass (Cordero et al., 2015), and gilthead sea bream (Jurado et al., 2015; Sanahuja and Ibarz, 2015). Differentially expressed proteins in skin mucus have been studied in response to aquaculture stressors, such as infection (Provan et al., 2013; Rajan et al., 2013; Valdenegro-Vega et al., 2014), handling or crowding (Easy and Ross, 2009, 2010; Pérez-Sánchez et al., 2017), and nutritional challenges (Micallef et al., 2017). Here, for the first time, we study how the mucus proteome responds to the environmental challenge of low temperatures, as in the cold season: one of the main concerns for gilthead sea bream aquaculture, reviewed in Ibarz et al. (2010a). Our study goes beyond a list of individual proteins with expressions that are modified by low temperatures, and attempts to elucidate the relationship of the modified proteins by building the interactome, or protein–protein interactions, using STRING tools (Szklarczyk et al., 2017). Despite initially proposed protein classification as structure, metabolism or protection related, the resulting interactome showed a central core strongly linking most of the differentially expressed proteins under cold conditions, and a satellite subset network including all the keratin forms detected together with periplakin and epiplakin proteins. From that central core of the cold interactome, four main subsets were obtained via enrichment analysis corresponding to GO groups with significance.

Within the “Response to stress” GO group (GO:0006950), consistent protein–protein interactions were reported for 12 proteins, indicating that defensive proteins, such as HSPs, TF, and PDIA3; metabolic proteins, such as PCNA, PPIF, and PSMA6; and structural proteins, such as GSN and COTL1, work together, also in skin mucus. Furthermore, whereas proteins with enzymatic activities (PDIA3, UBA1, PCNA or PSMA6) were down-regulated, the defensive proteins HSPs and TF were up-regulated. HSP forms and TF have been proposed as welfare biomarkers in mucus (Sanahuja and Ibarz, 2015), since the presence of chaperones has been related with mucus protein stability (Iq and Shu-Chien, 2011; Rajan et al., 2011) and the TF withholds iron and makes bacterial survival difficult, playing a role as an activator of fish macrophages (Stafford et al., 2001). Their up-regulation at low temperatures can probably be attributed to an increase of these unspecific and innate responses. All the proteins clustered as “Single-organism metabolic process” (GO:0044710) were under-expressed at low temperatures. In the skin mucus of sea bream, several proteins related to metabolism, and mainly with carbohydrate metabolism, were previously reported (Jurado et al., 2015; Sanahuja and Ibarz, 2015; Pérez-Sánchez et al., 2017). Once again, studies of challenges to different fish species have reported the increased presence of metabolic proteins in the skin mucus proteome (Provan et al., 2013; Rajan et al., 2013). For instance, a number of proteasome subunits and ubiquitin were up-regulated in fish mucus in response to infections (Bricknell et al., 2006; Rajan et al., 2013). In contrast, we attributed the current down-regulation of detected activities in mucus under cold conditions to overall metabolic depression (Ibarz et al., 2010b; Sánchez-Nuño et al., 2018a,b), which also affects exudation of these enzymes from epidermal cells. Thus, a lower presence of metabolic proteins exuded at low temperatures is also an indicator of lower metabolism in skin, and a putative lower capacity to cope with further challenges, such as infections.

Another interactome subset was linked to “Interspecies interaction between organisms” (GO:0044419), which included mainly up-regulated mucus proteins. This interactome subset evidenced a favorable condition for bacteria adhesion at low temperatures due to changes in the proteome. *Hsp70* may be a stress-induced surface adhesin, mediating sulfatide recognition, that could be used by bacteria to facilitate surface adhesion (Valizadeh et al., 2017), just as lectin types are used by infectious organisms to bind with complementary host structures (Acord et al., 2005). Septins, together with actin, are increasingly recognized as playing important roles in bacterial entry into host cells (Mostowy et al., 2009) including those of fish (Willis et al., 2016). Meanwhile, 40S ribosomal protein is required for an adhesion process that depends upon both cell–cell and cell–substrate adherence of several fungal pathogens (Kim et al., 2010); although in fish, greater amounts of ribosomal proteins in skin mucus were reported in response to infection (Esteban, 2012). Epiplakin and periplakin, as desmosome components, and keratin-8, seem to work together in maintaining tissue integrity, mainly in keratinocyte layers (Long et al., 2006). Their up-regulation was observed in the present study, which is a signal of a putative response to block bacterial entry or to regulate epithelial cell turnover in chronic low temperature conditions.

Interestingly, the interactome approach resulted in a group of proteins being clustered in the “Transport” GO-group (GO:0006810), and all were over-expressed. It is well-known that mucus cells in fish epidermis package their products in secreting vesicles and release the contents through exocytosis processes (Long et al., 2013), similarly to the mucus-secreting cells of mammals (Verdugo, 1990). However, the molecular mechanisms underlying the synthesis and release of bioactive mucus products, and the responses of mucus cells to environmental stressors or pathogens, remain largely unknown. Our results would indicate that, in spite of overall depression under cold conditions, fish made efforts to maintain the rate of mucus secretion at low temperatures, because mucus turnover (the balance between continuous secretion and replacement) is crucial to prevent potential infections (Esteban, 2012). However, further studies should focus on mucus turnover and renewal under natural and challenged conditions, considering both epidermal cell activities, and mucus properties, and composition.

Finally, the proteome map of gilthead sea bream skin mucus at low temperatures showed a number of fragments or cleaved proteins, mainly keratin forms. Recently, interest in the presence of cleaved keratins has increased due to their putative antimicrobial function as membrane pore-forming peptides in mammals (Tam et al., 2012). The so-called KDAMPs are produced by proteolysis via extracellular proteases. In fish, little information on the roles of keratin as antimicrobial peptides is available. Different reports have shown that keratins from skin mucus also possess anti-bacterial activity, owing to their pore-forming properties (Molle et al., 2008; Rajan et al., 2011). For gilthead sea bream, Sanahuja and Ibarz (2015) noted the presence of keratin fragments in the skin mucus proteome and Pérez-Sánchez et al. (2017) also revealed by Western blot the presence of several forms, with different MWs, of cytokeratin-8 as a product of proteolytic activity. In accordance with that, in the current study we identified two bands for cytokeratin-8, which corresponded to the proteome presence of a small fragment (around 14 kDa). An increasing number of antimicrobial peptides in fish mucus are found to be derived by proteolysis from larger proteins with other known functions, such as ribosomal proteins (Cho et al., 2002b). It seems that matrix metalloproteinase 2 is involved in the regulation of that proteolysis in mucus, activating cathepsin forms. Thus, up-regulation of the specific metalloprotease activity detected by zymography together with higher concentrations of ribosomal and keratin fragments in skin mucus suggest an increased innate defense via new antimicrobial peptides during chronic cold in sea bream. This is the first approach using 2D-SDS-PAGE coupled to LC-MS/MS analysis to report a number of differentially expressed protein fragments in skin mucus. As it would be difficult to identify fragments of native proteins by the respective antibodies, as occurred here with the different spots corresponding to actin, further approaches will be necessary to focus on those fragments, the sequence to be identified and the antimicrobial role attributed to them.

## Conclusion

Skin mucus studies have been shown to be a powerful tool to devise putative bioindicators of fish welfare and physiological status via non-invasive methods. Here, we demonstrate that the skin mucus proteome also reflects the reported overall depression of gilthead sea bream metabolism and immune response at low temperatures. Under a chronic cold challenge, the capacity of fish to exude protective components to the main external fish barrier was altered, reducing mainly proteins related to enzymatic activity. However, alternative innate defenses appeared, such as HSPs, transferrin or lower-molecular-weight antimicrobial peptides. Additionally, some mucus proteins related to pathogen adhesion were increased at low temperatures, which would favor infection processes. In view of present results, further studies are necessary to enhance understanding of the impact of low environmental temperatures on the acute or short-term performance of host–pathogen systems, as well as during temperature recovery. Specifically, it would be advantageous to elucidate the underlying mucosal defense mechanisms that result in host mortality when fish suffer cold stress under farm conditions.

## Author Contributions

IS, LF-A, SS-N, BO-G, and AI performed the experiments. IS and AI designed the trial. All authors revised the manuscript, agreed to be accountable for the content of the work, and agreed to be listed and approved the submitted version of the manuscript.

### Conflict of Interest Statement

The authors declare that the research was conducted in the absence of any commercial or financial relationships that could be construed as a potential conflict of interest.

## References

[B1] Abram Q. H. Dixon B. Katzenback B. A. (2017). Impacts of low temperature on the teleost immune system. Biology 6:39. 10.3390/biology6040039 29165340 PMC5745444

[B2] Acord J. Maskell J. Sefton A. (2005). A rapid microplate method for quantifying inhibition of bacterial adhesion to eukaryotic cells. J. Microbiol. Methods 60, 55–62. 10.1016/j.mimet.2004.08.011 15567225

[B3] Alarcón F. J. Díaz M. Moyano F. J. Abellán E. (1998). Characterization and functional properties of digestive proteases in two sparids; gilthead seabream (*Sparus aurata*) and common dentex (*Dentex dentex*). Fish Physiol. Biochem. 19, 257–267. 10.1023/A:1007717708491

[B4] Aranishi F. Mano N. Hirose H. (1998). Fluorescence localization of epidermal cathepsins L and B in the Japanese eel. Fish Physiol. Biochem. 19, 205–209. 10.1023/A:1007779600183

[B5] Aranishi F. Nakane M. (1997). Epidermal proteases of the Japanese eel. Fish Physiol. Biochem. 16, 471–478. 10.1023/A:1007736804243

[B6] Benhamed S. Guardiola F. A. Mars M. Esteban M. Á. (2014). Pathogen bacteria adhesion to skin mucus of fishes. Vet. Microbiol. 171, 1–12. 10.1016/j.vetmic.2014.03.008 24709124

[B7] Benjamini Y. Hochberg Y. (1995). Controlling the false discovery rate: a practical and powerful approach to multiple testing. *J. Roy. Statist. Soc*. B. 57, 289–300.

[B8] Bradford M. (1976). A rapid and sensitive method for the quantitation of microgram quantities of protein utilizing the principle of protein-dye binding. Anal. Biochem. 72, 248–254. 10.1016/0003-2697(76)90527-3 942051

[B9] Braun R. Arnesen J. A. Rinne A. Hjelmeland K. (1990). Immunohistological localization of trypsin in mucus-secreting cell layers of Atlantic salmon, *Salmo salar* L. J. Fish Dis. 13, 233–238. 10.1111/j.1365-2761.1990.tb00778.x

[B10] Bricknell I. R. Bron J. E. Bowden T. J. (2006). Diseases of gadoid fish in cultivation: a review. ICES J. Mar. Sci. 63, 253–266. 10.1016/j.icesjms.2005.10.009

[B11] Caruso G. Denaro M. G. Caruso R. Mancari F. Genovese L. Maricchiolo G. (2011). Response to short term starvation of growth, haematological, biochemical and non-specific immune parameters in European sea bass (*Dicentrarchus labrax*) and blackspot sea bream (*Pagellus bogaraveo*). Mar. Environ. Res. 72, 46–52. 10.1016/j.marenvres.2011.04.005 21664688

[B12] Cho J. H. Park I. Y. Kim H. S. Lee W. T. Kim M. S. Kim S. C. (2002a). Cathepsin D produces antimicrobial peptide parasin I from histone H2A in the skin mucosa of fish. FASEB J. 16, 429–431. 10.1096/fj.01-0736fje 11821259

[B13] Cho J. H. Park I. Y. Kim M. S. Kim S. C. (2002b). Matrix metalloproteinase 2 is involved in the regulation of the antimicrobial peptide parasin I production in catfish skin mucosa. FEBS Lett. 531, 459–463. 10.1016/S0014-5793(02)03584-6 12435593

[B14] Chong K. Ying T. S. Foo J. Jin L. T. Chong A. (2005). Characterisation of proteins in epidermal mucus of discus fish (*Symphusodon* spp.) during parental phase. Aquaculture 249, 469–476. 10.1016/j.aquaculture.2005.02.045

[B15] Cone R. A. (2009). Barrier properties of mucus. Adv. Drug Deliv. Rev. 61, 75–85. 10.1016/j.addr.2008.09.008 19135107

[B16] Cordero H. Brinchmann M. F. Cuesta A. Esteban M. A. (2017). Chronic wounds alter the proteome profile in skin mucus of farmed gilthead seabream. BMC Genomics 18:939. 10.1186/s12864-017-4349-3 29197330 PMC5712093

[B17] Cordero H. Morcillo P. Cuesta A. Brinchmann M. F. Esteban M. A. (2015). Differential proteome profile of skin mucus of gilthead seabream (*Sparus aurata*) after probiotic intake and/or overcrowding stress. J. Proteomics 132, 41–50. 10.1016/j.jprot.2015.11.017 26617323

[B18] De Mercado E. Larrán A. M. Pinedo J. Tomás-Almenar C. Hurst T. P. (2018). Skin mucous: a new approach to assess stress in rainbow trout. Aquaculture 484, 90–97. 10.1016/j.aquaculture.2017.10.031

[B19] Easy R. H. Ross N. W. (2009). Changes in Atlantic salmon (*Salmo salar*) epidermal mucus protein composition profiles following infection with sea lice (*Lepeophtheirus salmonis*). Comp. Biochem. Physiol. Part D Genomics Proteomics 4, 159–167. 10.1016/j.cbd.2009.02.001 20403764

[B20] Easy R. H. Ross N. W. (2010). Changes in Atlantic salmon *Salmo salar* mucus components following short- and long-term handling stress. J. Fish Biol. 77, 1616–1631. 10.1111/j.1095-8649.2010.02796.x 21078022

[B21] Ellis A. E. (2001). Innate host defense mechanisms of fish against viruses and bacteria. Dev. Comp. Immunol. 25, 827–839. 10.1016/S0145-305X(01)00038-6 11602198

[B22] Esteban M. A. (2012). An overview of the immunological defenses in fish skin. ISRN Immunol. 2012:853470 10.5402/2012/853470

[B23] Fernández-Alacid L. Sanahuja I. Ordóñez-Grande B. Sánchez-Nuño S. Herrera M. Ibarz A. (2019). Skin mucus metabolites and cortisol in meagre fed acute stress-attenuating diets: correlations between plasma and mucus. Aquaculture 499, 185–194. 10.1016/J.AQUACULTURE.2018.09.039

[B24] Fernández-Alacid L. Sanahuja I. Ordóñez-Grande B. Sánchez-Nuño S. Viscor G. Gisbert E. (2018). Skin mucus metabolites in response to physiological challenges: a valuable non-invasive method to study teleost marine species. Sci. Total Environ. 644, 1323–1335. 10.1016/j.scitotenv.2018.07.083 30743845

[B25] Firth K. J. Johnson S. C. Ross N. W. (2000). Characterization of proteases in the skin mucus of Atlantic salmon (*Salmo salar*) infected with the salmon louse (*Lepeophtheirus salmonis*) and in whole-body louse homogenate. J. Parasitol. 86, 1199–1205. 10.1645/0022-3395(2000)086[1199:COPITS]2.0.CO;2 11191891

[B26] García-Carreño F. L. Dimes L. E. Haard N. F. (1993). Substrate-gel electrophoresis for composition and molecular weight of proteinases or proteinaceous proteinase inhibitors. Anal. Biochem. 214, 65–69. 10.1006/abio.1993.1457 8250256

[B27] García-Meilán I. Valentín J. M. Fontanillas R. Gallardo M. A. (2013). Different protein to energy ratio diets for gilthead sea bream (*Sparus aurata*): effects on digestive and absorptive processes. Aquaculture 412–413, 1–7. 10.1016/j.aquaculture.2013.06.031

[B28] Guardiola F. A. Cuesta A. Abellán E. Meseguer J. Esteban M. A. (2014a). Comparative analysis of the humoral immunity of skin mucus from several marine teleost fish. Fish Shellfish Immunol. 40, 24–31. 10.1016/j.fsi.2014.06.018 24972341

[B29] Guardiola F. A. Cuesta A. Arizcun M. Meseguer J. Esteban M. A. (2014b). Comparative skin mucus and serum humoral defence mechanisms in the teleost gilthead seabream (*Sparus aurata*). Fish Shellfish Immunol. 36, 545–551. 10.1016/j.fsi.2014.01.001 24412437

[B30] Guardiola F. A. Dioguardi M. Parisi M. G. Trapani M. R. Meseguer J. Cuesta A.. (2015). Evaluation of waterborne exposure to heavy metals in innate immune defences present on skin mucus of gilthead seabream (*Sparus aurata*). Fish Shellfish Immunol. 45, 112–123. 10.1016/j.fsi.2015.02.010 25700783

[B31] Hjelmeland K. Christie M. Raa J. (1983). Skin mucus protease from rainbow trout, *Salmo gairdneri* Richardson, and its biological significance. J. Fish Biol. 23, 13–22. 10.1111/j.1095-8649.1983.tb02878.x

[B32] Hurst T. P. (2007). Causes and consequences of winter mortality in fishes. J. Fish Biol. 71, 315–345. 10.1111/j.1095-8649.2007.01596.x

[B33] Ibarz A. Blasco J. Gallardo M. A. Fernández-Borràs J. (2010b). Energy reserves and metabolic status affect the acclimation of gilthead sea bream (*Sparus aurata*) to cold. Comp. Biochem. Physiol. A Mol. Integr. Physiol. 155, 319–326. 10.1016/j.cbpa.2009.11.012 19931633

[B34] Ibarz A. Padrós F. Gallardo M. A. Fernández-Borràs J. Blasco J. Tort L. (2010a). Low-temperature challenges to gilthead sea bream culture: review of cold-induced alterations and “Winter Syndrome.” Rev. Fish Biol. Fish. 20, 539–556. 10.1007/s11160-010-9159-5

[B35] Ingram G. A. (1980). Substances involved in the natural resistance of fish to infection–a review. J. Fish Biol. 16, 23–60. 10.1111/j.1095-8649.1980.tb03685.x

[B36] Iq K. C. Shu-Chien A. C. (2011). Proteomics of buccal cavity mucus in female tilapia fish (*Oreochromis* spp.): a comparison between parental and non-parental fish. PLoS ONE 6:e18555. 10.1371/journal.pone.0018555 21533134 PMC3080365

[B37] Jung T. S. del Castillo C. S. Javaregowda P. K. Dalvi R. S. Nho S. W. Park S. B.. (2012). Seasonal variation and comparative analysis of non-specific humoral immune substances in the skin mucus of olive flounder (*Paralichthys olivaceus*). Dev. Comp. Immunol. 38, 295–301. 10.1016/j.dci.2012.06.005 22750133

[B38] Jurado J. Fuentes-Almagro C. A. Guardiola F. A. Cuesta A. Esteban M. Á. Prieto-Álamo M.-J. (2015). Proteomic profile of the skin mucus of farmed gilthead seabream (*Sparus aurata*). J. Proteomics 120, 21–34. 10.1016/j.jprot.2015.02.019 25753121

[B39] Kim S. W. Joo Y. J. Kim J. (2010). Asc1p, a ribosomal protein, plays a pivotal role in cellular adhesion and virulence in *Candida albicans*. J. Microbiol. 48, 842–848. 10.1007/s12275-010-0422-1 21221944

[B40] Loganathan K. Arulprakash A. Prakash M. Senthilraja P. (2013). Lysozyme, protease, alkaline phosphatase and esterase activity of epidermal skin mucus of freshwater snake head fish *Channa striatus*. Int. J. Res. Pharm. Biosci. 3, 17–20.

[B41] Long H. A. Boczonadi V. McInroy L. Goldberg M. Maatta A. (2006). Periplakin-dependent re-organisation of keratin cytoskeleton and loss of collective migration in keratin-8-downregulated epithelial sheets. J. Cell Sci. 119, 5147–5159. 10.1242/jcs.03304 17158917

[B42] Long Y. Li Q. Zhou B. Song G. Li T. Cui Z. (2013). *De novo* assembly of mud loach (*Misgurnus anguillicaudatus*) skin transcriptome to identify putative genes involved in immunity and epidermal mucus secretion. PLoS ONE 8:e56998. 10.1371/journal.pone.0056998 23437293 PMC3577766

[B43] Mayer L. (2003). Mucosal immunity. Pediatrics 111, 1595–1600. 10.1542/peds.111.6.S2.1595 12777598

[B44] McCawley L. J. Matrisian L. M. (2001). Matrix metalloproteinases: they're not just for matrix anymore! Curr. Opin. Cell Biol. 13, 534–540. 10.1016/S0955-0674(00)00248-9 11544020

[B45] Micallef G. Cash P. Fernandes J. M. O. Rajan B. Tinsley J. W. Bickerdike R.. (2017). Dietary yeast cell wall extract alters the proteome of the skin mucous barrier in atlantic salmon (*Salmo salar*): increased abundance and expression of a calreticulin-like protein. PLoS ONE 12:e0169075. 10.1371/journal.pone.0169075 28046109 PMC5207756

[B46] Molle V. Campagna S. Bessin Y. Ebran N. Saint N. Molle G. (2008). First evidence of the pore-forming properties of a keratin from skin mucus of rainbow trout (*Oncorhynchus mykiss*, formerly *Salmo gairdneri*). Biochem. J. 411, 33–40. 10.1042/BJ20070801 18052924

[B47] Mostowy S. Tham T. N. Danckaert A. Guadagnini S. Boisson-Dupuis S. Pizarro-Cerdá J.. (2009). Septins regulate bacterial entry into host cells. PLoS ONE 4:e4196. 10.1371/journal.pone.0004196 19145258 PMC2626286

[B48] Moyano F. J. Díaz M. Alarcón F. J. Sarasquete M. C. (1996). Characterization of digestive enzyme activity during larval development of gilthead seabream (*Sparus aurata*). Fish Physiol. Biochem. 15, 121–130. 10.1007/BF01875591 24194085

[B49] Nagashima Y. Kikuchi N. Shimakura K. Shiomi K. (2003). Purification and characterization of an antibacterial protein in the skin secretion of rockfish *Sebastes schlegeli*. Comp. Biochem. Physiol. C Toxicol. Pharmacol. 136, 63–71. 10.1016/S1532-0456(03)00174-1 14522599

[B50] Nigam A. K. Kumari U. Mittal S. Mittal A. K. (2012). Comparative analysis of innate immune parameters of the skin mucous secretions from certain freshwater teleosts, inhabiting different ecological niches. Fish Physiol. Biochem. 38, 1245–1256. 10.1007/s10695-012-9613-5 22350522

[B51] Parry R. M. Chandan R. C. Shahani K. M. (1965). A rapid and sensitive assay of muramidase. Exp. Biol. Med. 119, 384–386. 10.3181/00379727-119-30188 14328897

[B52] Patel D. M. Brinchmann M. F. (2017). Skin mucus proteins of lumpsucker (*Cyclopterus lumpus*). Biochem. Biophys. Rep. 9, 217–225. 10.1016/j.bbrep.2016.12.016 28956008 PMC5614610

[B53] Pérez-Sánchez J. Terova G. Simó-Mirabet P. Rimoldi S. Folkedal O. Calduch-Giner J. A.. (2017). Skin mucus of gilthead sea bream (*Sparus aurata* L.). Protein mapping and regulation in chronically stressed fish. Front. Physiol. 8:34. 10.3389/fphys.2017.00034 28210224 PMC5288811

[B54] Provan F. Jensen L. B. Uleberg K. E. Larssen E. Rajalahti T. Mullins J.. (2013). Proteomic analysis of epidermal mucus from sea lice-infected Atlantic salmon, *Salmo salar* L. J. Fish Dis. 36, 311–321. 10.1111/jfd.12064 23305410

[B55] Rajan B. Fernandes J. M. O. Caipang C. M. A Kiron V. Rombout J. H. W. M. Brinchmann M. F. (2011). Proteome reference map of the skin mucus of Atlantic cod (*Gadus morhua*) revealing immune competent molecules. Fish Shellfish Immunol. 31, 224–231. 10.1016/j.fsi.2011.05.006 21609766

[B56] Rajan B. Lokesh J. Kiron V. Brinchmann M. F. (2013). Differentially expressed proteins in the skin mucus of Atlantic cod (*Gadus morhua*) upon natural infection with *Vibrio anguillarum*. BMC Vet. Res. 9:103. 10.1186/1746-6148-9-103 23672475 PMC3666997

[B57] Rakers S. Niklasson L. Steinhagen D. Kruse C. Schauber J. Sundell K.. (2013). Antimicrobial peptides (AMPs) from fish epidermis: perspectives for investigative dermatology. J. Invest. Dermatol. 133, 1140–1149. 10.1038/jid.2012.503 23407389

[B58] Rivals I. Personnaz L. Taing L. Potier M. C. (2007). Enrichment or depletion of a GO category within a class of genes: which test? Bioinformatics 23, 401–407. 10.1093/bioinformatics/btl633 17182697

[B59] Ross N. W. Firth K. J. Wang A. Burka J. F. Johnson S. C. (2000). Changes in hydrolytic enzyme activities of naive Atlantic salmon *Salmo salar* skin mucus due to infection with the salmon louse *Lepeophtheirus salmonis* and cortisol implantation. Dis. Aquat. Organ. 41, 43–51. 10.3354/dao041043 10907138

[B60] Sanahuja I. Ibarz A. (2015). Skin mucus proteome of gilthead sea bream: a non-invasive method to screen for welfare indicators. Fish Shellfish Immunol. 46, 426–435. 10.1016/j.fsi.2015.05.056 26134830

[B61] Sánchez-Nuño S. Eroldogan O. Sanahuja I. Özşahinoglu I. Blasco J. Fernández-Borràs J. (2018a). Cold-induced growth arrest in gilthead sea bream *Sparus aurata*: metabolic reorganisation and recovery. Aquac. Environ. Interact. 10, 511–528. 10.3354/aei00286

[B62] Sánchez-Nuño S. Sanahuja I. Fernández-Alacid L. Ordóñez-Grande B. Fontanillas R. Fernández-Borràs J.. (2018b). Redox challenge in a cultured temperate marine species during low temperature and temperature recovery. Front. Physiol. 9:923. 10.3389/fphys.2018.00923 30065660 PMC6056653

[B63] Santigosa E. Sánchez J. Médale F. Kaushik S. Pérez-Sánchez J. Gallardo M. A. (2008). Modifications of digestive enzymes in trout (*Oncorhynchus mykiss*) and sea bream (*Sparus aurata*) in response to dietary fish meal replacement by plant protein sources. Aquaculture 282, 68–74. 10.1016/j.aquaculture.2008.06.007

[B64] Silva T. S. da Costa A. M. R. Conceição L. E. C. Dias J. P. Rodrigues P. M. L. Richard N. (2014). Metabolic fingerprinting of gilthead seabream (*Sparus aurata*) liver to track interactions between dietary factors and seasonal temperature variations. PeerJ 2:e527. 10.7717/peerj.527 25210655 PMC4157298

[B65] Stafford J. L. Neumann N. F. Belosevic M. (2001). Products of proteolytic cleavage of transferrin induce nitric oxide response of goldfish macrophages. Dev. Comp. Immunol. 25, 101–115. 10.1016/S0145-305X(00)00048-3 11113281

[B66] Szklarczyk D. Morris J. H. Cook H. Kuhn M. Wyder S. Simonovic M.. (2017). The STRING database in 2017: quality-controlled protein-protein association networks, made broadly accessible. Nucleic Acids Res. 45, D362–D368. 10.1093/nar/gkw937 27924014 PMC5210637

[B67] Tam C. Mun J. J. Evans D. J. Fleiszig S. M. J. (2012). Cytokeratins mediate epithelial innate defense through their antimicrobial properties. J. Clin. Invest. 122, 3665–3677. 10.1172/JCI64416 23006328 PMC3461926

[B68] Tkachenko A. Da Silva L. Hearne J. Parveen S. Waguespack Y. (2013). An assay to screen bacterial adhesion to mucus biomolecules. Lett. Appl. Microbiol. 56, 79–82. 10.1111/lam.12003 23020180

[B69] Toranzo A. E. Magariños B. Romalde J. L. (2005). A review of the main bacterial fish diseases in mariculture systems. Aquaculture 246, 37–61. 10.1016/j.aquaculture.2005.01.002

[B70] Tort L. Padrós F. Rotllant J. Crespo S. (1998a). Winter syndrome in the gilthead sea bream *Sparus aurata*. Immunological and histopathological features. Fish Shellfish Immunol. 8, 37–47. 10.1006/fsim.1997.0120

[B71] Tort L. Rotllant J. Rovira L. (1998b). Immunological suppression in gilthead sea bream *Sparus aurata* of the North-West Mediterranean at low temperatures. Comp. Biochem. Physiol. Part A 120, 175–179. 10.1016/S1095-6433(98)10027-2

[B72] Valdenegro-Vega V. A. Crosbie P. Bridle A. Leef M. Wilson R. Nowak B. F. (2014). Differentially expressed proteins in gill and skin mucus of Atlantic salmon (*Salmo salar*) affected by amoebic gill disease. Fish Shellfish Immunol. 40, 69–77. 10.1016/j.fsi.2014.06.025 24979223

[B73] Valizadeh A. Ir P. Khosravi A. (2017). Investigating the role of thermal shock protein (Dank) HSP70 in bacteria. *J. Bacteriol*. Mycol. 4:1055.

[B74] Vargas-Chacoff L. Arjona F. J. Ruiz-Jarabo I. Páscoa I. Gonçalves O. Martín Del Río M. P. (2009). Seasonal variation in osmoregulatory and metabolic parameters in earthen pond-cultured gilthead sea bream *Sparus auratus*. Aquac. Res. 40, 1279–1290. 10.1111/j.1365-2109.2009.02226.x

[B75] Verdugo P. (1990). Goblet cells secretion and mucogenesis. Annu. Rev. Physiol. 52, 157–176. 10.1146/annurev.physiol.52.1.157 2184755

[B76] Willis A. Mazon-Moya M. Mostowy S. (2016). Investigation of septin biology *in vivo* using zebrafish. Methods Cell Biol. 136, 221–241. 10.1016/bs.mcb.2016.03.019 27473912

[B77] Woessner J. F. Jr. (1991). Matrix metalloproteinases and their inhibitors in connective tissue remodeling. FASEB J. 5, 2145–2154. 10.1096/fasebj.5.8.1850705 1850705

