# Supplementary material for: Chronic Cold Stress Alters the Skin Mucus Interactome in a Temperate Fish Model
Source: Front Physiol. 2019 Jan 11;9:1916. doi: 10.3389/fphys.2018.01916 (PMC6336924; doi:10.3389/fphys.2018.01916)

**Supplementary Figure 1. Location of differentially expressed proteins due to chronic cold challenge in the skin mucus proteome 2D-map**. More than 1200 protein spots were detected in the mucus proteome of all the samples after 2Dgel staining. In primary matched sets, a representative “Master” gel was obtained for the Warm condition and the 300 spots with higher normalized intensity were further analysed for their differential expression between the Warm and Cold conditions. Green spots are over-expressed due to the cold. Pink spots were under-expressed due to the cold. After a cleaning process, the protein extract was separated on 24 cm non-linear pH 3-10 IPG strips, followed by separation using 12.5% SDS-PAGE. Numbers indicate the Spot-ID according to proteins listed in Tables 1 and 2.


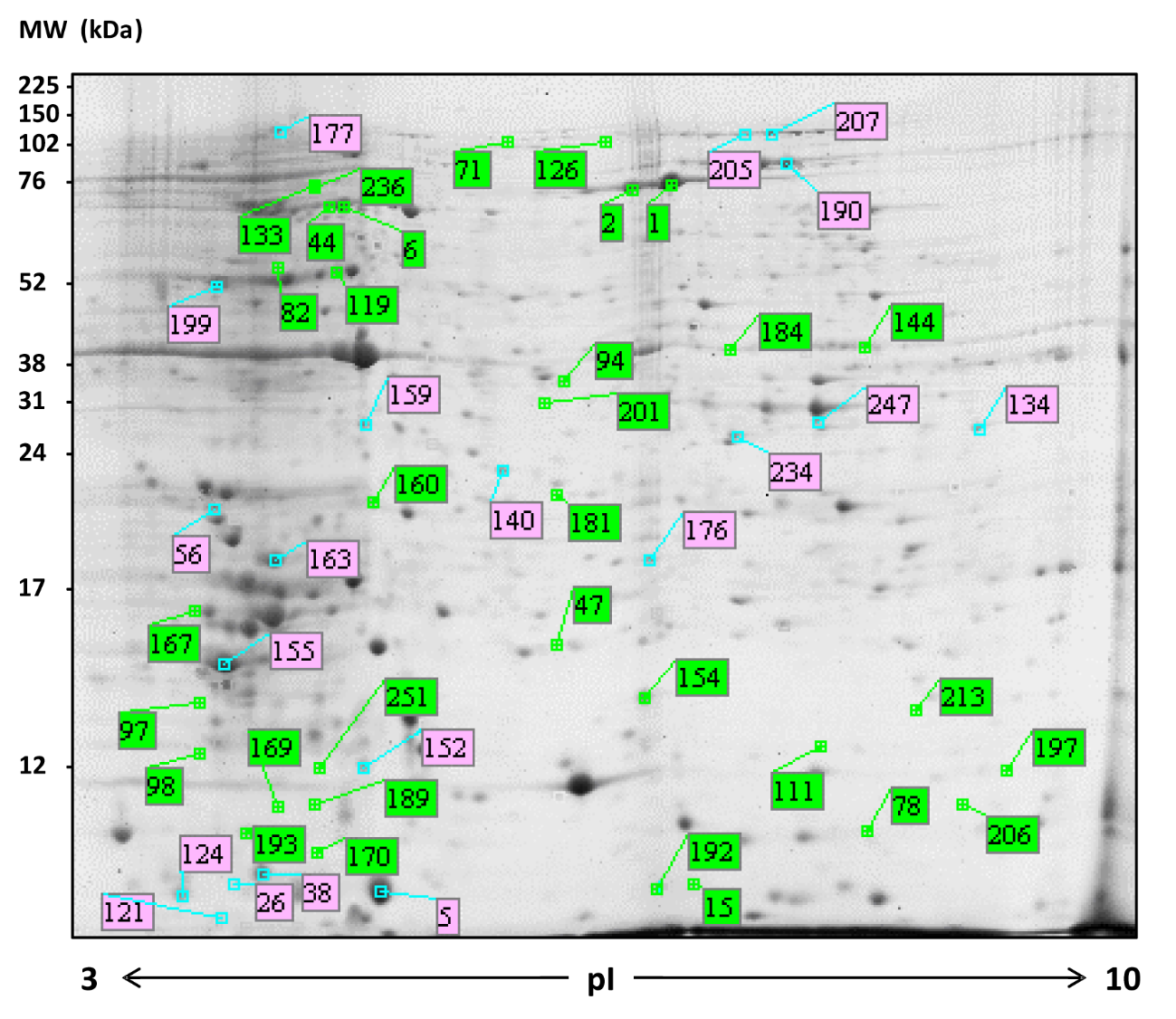

Supplement: Supplementary file 1 [file Table_1.DOCX]
