# Supplementary material for: Chronic Cold Stress Alters the Skin Mucus Interactome in a Temperate Fish Model
Source: Front Physiol. 2019 Jan 11;9:1916. doi: 10.3389/fphys.2018.01916 (PMC6336924; doi:10.3389/fphys.2018.01916)
Supplement: Supplementary file 2 [file Table_2.DOCX]

**Supplementary Figure 2**. Digestive Bands quantification


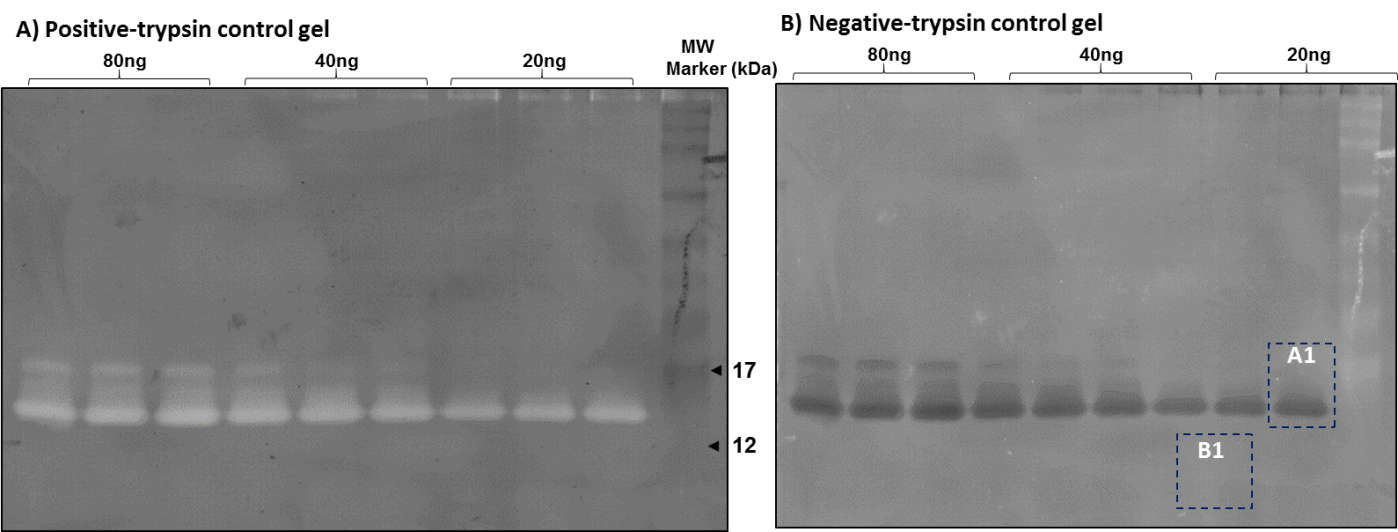
Trypsin reference gel was performed (Figure A) with known trypsin amounts per triplicate (80ng, 40 ng and 20 ng or trypsin loaded in each lane). Gels are scanned in an ImageScanner III (Epson J181A) and caseinolytic bands are identified at the expected MW of 15kDa. Negative image from the gel is obtained to show the intensity for the corresponding caseinolytic band (Fig. B). The relative digestion units for each trypsin amount were obtained from the dotted area of each lane (subtracting corresponding background intensity, dotted area B1). Under current conditions (2h of casein incubation, see M&M) the linearity of the trypsin digestion was conserved at trypsin amount below 80ng (Fig. C).


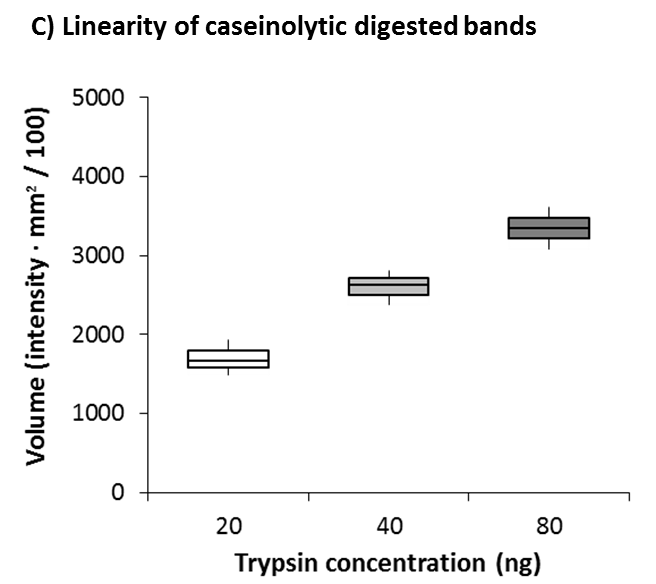


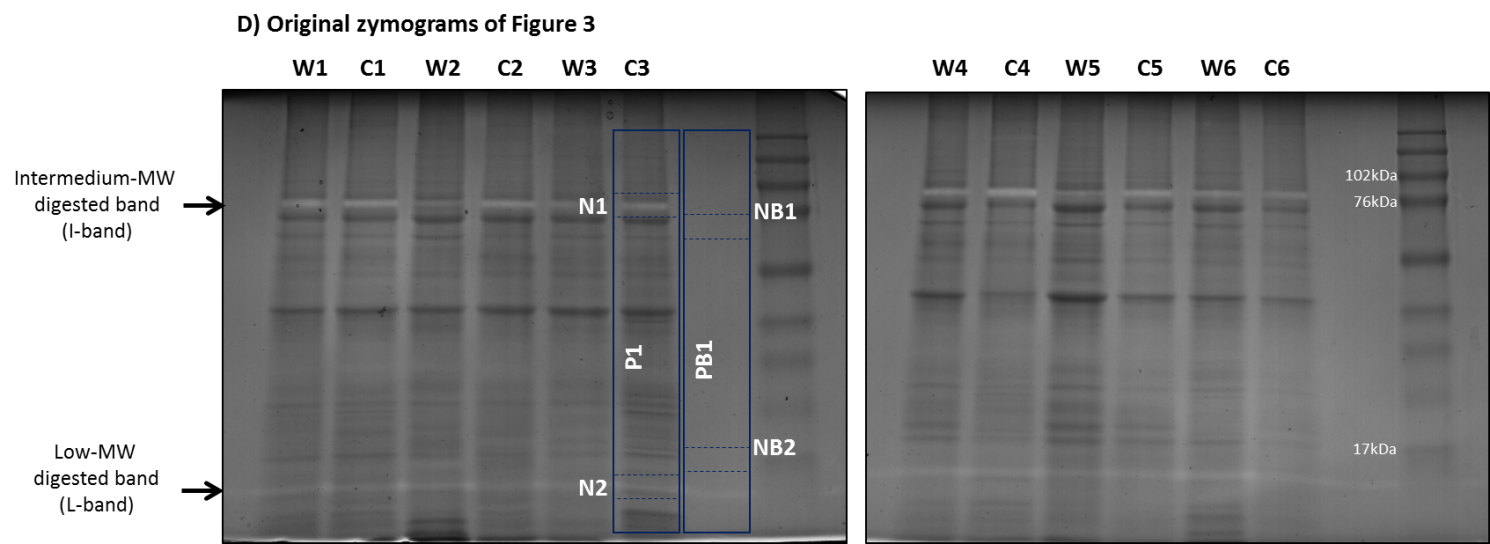
The relative digestion units for each caseinolytic band showed in Figure 3 were obtained by the relation between the band quantification and the total lane intensity. Digestion band intensity was calculated as arbitrary units of casein digestion capacity (Fig. D): the area intensity of each specific digested band (dotted areas, N1 for I-band and N2 for L-band dotted areas, respectively and subtracting corresponding background intensity NB1 and NB2) via the negative image (not showed), was related to the total intensity of the respective undigested lane (P1 lane area previously removing the background, PB1 lane area).
