# Supplementary material for: Chronic Cold Stress Alters the Skin Mucus Interactome in a Temperate Fish Model
Source: Front Physiol. 2019 Jan 11;9:1916. doi: 10.3389/fphys.2018.01916 (PMC6336924; doi:10.3389/fphys.2018.01916)
Supplement: Supplementary file 3 [file Table_3.DOCX]

| **Supplementary Table 1.** Network stats and functional enrichments of skin mucus interactomes. | | | | | | |
| --- | --- | --- | --- | --- | --- | --- |
|  | Fig 1A | Fig 1B | Fig 1C | Fig 1D | Fig 1E |  |
| Interactome networks | Global Interactome | Response to stress | Metabolic process | Inter-species interaction | Transport |  |
| Network stats^1^ |  |  |  |  |  |  |
| *Average local clustering coefficient* | 0.447 | 0.536 | 0.397 | 0.515 | 0.763 |  |
| *PPI enrichment p-value* | 9.83e-05 | 0.0244 | 0.00258 | 0.0421 | 5.44e-05 |  |
| Functional enrichment^2^ |  |  |  |  |  |  |
| *Pathway ID* |  | GO:0006950 | GO:0044710 | GO:0044419 | GO:0006810 |  |
| *Pathway description* |  | Response to stress | Single-organism metabolic process | Interspecies interaction between organisms | Transport |  |
| *Count in gene set* |  | 18 | 11 | 7 | 10 |  |
| *False discovery rate* |  | 3.35e-07 | 5.82e-03 | 1.55e-04 | 0.0111 |  |
| 1. Network stats: Clustering coefficients correspond to a measure of how connected the nodes in the network are. Highly connected networks have high values (between 0 and 1). Low PPI (protein-protein interaction) enrichment p-values indicate that the nodes are not random and that the observed number of edges is significant. Both indices are obtained from data analysis by STRING Program v10.5. 2. Functional enrichments of each cluster (Figure 1) have been performed using the protein groups from Table 2. Pathway ID and description matched with those reported in Table 2, being “count in gene set” the number of proteins included with their “false discovery rate”. | | | | | |  |
